# DPPH Measurements and Structure—Activity Relationship Studies on the Antioxidant Capacity of Phenols

**DOI:** 10.3390/antiox13030309

**Published:** 2024-03-01

**Authors:** Moeka Yamauchi, Yukino Kitamura, Haruka Nagano, Junya Kawatsu, Hiroaki Gotoh

**Affiliations:** Department of Chemistry and Life Science, Yokohama National University, Hodogaya-ku, Yokohama 240-8501, Japan; yamauchi-moeka-xc@ynu.jp (M.Y.);

**Keywords:** DPPH, ionization potential, antioxidant capacity, phenol, SAR, food informatics

## Abstract

The consumption of foods that are high in antioxidant capacity is believed to contribute to good health. Moreover, the addition of highly antioxidant compounds to foods is believed to prevent food deterioration. Among the known antioxidants in food, phenols have been identified as the primary antioxidants. The 2,2-diphenyl-1-picrylhydrazyl (DPPH) assay is a simple, inexpensive, and rapid method widely used to evaluate the antioxidant capacity. Although the results of the DPPH assay depend on conditions such as the reaction time and concentration, the experimental conditions have not been standardized. Further, previous research that compared the antioxidant capacity determined through the DPPH assay largely focused on the differences in the specific substructures of approximately several dozen compounds. In this study, we conducted DPPH assays on 169 phenols under the same experimental conditions and summarized the correlation between their structures and activity. This DPPH assay study is the first single-laboratory investigation of the largest number of components in terms of their Trolox equivalent antioxidant capacities. Further, the analysis method was reproduced in an interlaboratory collaborative study, enabling its application in the reproduction and comparison of measurements in other laboratories.

## 1. Introduction

Reactive oxygen species are highly reactive, and when produced in excess amounts due to redox imbalance in the body, they attack biomolecules and may be the root cause of various diseases [1,2]. Antioxidants slow or deter oxidation when acting in small amounts on substrates being oxidized [3]. An inverse association has been reported between the dietary intake of antioxidant-rich foods and the prevalence of chronic diseases [4]. Antioxidants are also used as food additives to prevent food oxidation and deterioration [5]. The main antioxidants used for this purpose are polyphenols. For example, a relationship [6] has been demonstrated between the total polyphenol content and antioxidant capacity of hops, beer [7], and plant extracts. A correlation has also been established between the total polyphenol content and antioxidant capacity of vegetables and fruits commonly consumed in Japan [8].

The 2,2-diphenyl-1-picrylhydrazyl (DPPH) assay measures the scavenging capacity of antioxidants against the free radical DPPH [9]. Various in vitro assays have been proposed for estimating the antioxidant capacity, such as the oxygen radical absorbance capacity (ORAC), 2,2′-azinobis-3-ethylbenzothiazoline-6-sulfonic acid, ferric reducing antioxidant power, and superoxide dismutase assays. Nevertheless, the DPPH assay is widely preferred because it is inexpensive, simple, and rapid [10]. Although the antioxidant capacity measurements in such in vitro studies were acknowledged to have conceptual and technical limitations [11], such studies are useful for confirming whether a compound possesses antioxidant capacity [12,13].

**Figure 1:**
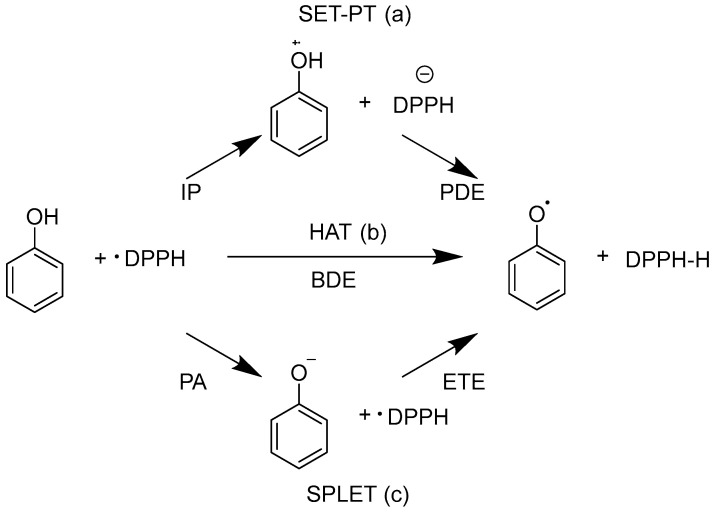
Three possible antioxidant mechanisms for the reaction of 2,2-diphenyl-1-picrylhydrazyl (DPPH) with phenol: (**a**) single electron transfer followed by proton transfer (SET-PT); (**b**) single-step hydrogen atom transfer (HAT); and (**c**) sequential proton loss electron transfer (SPLET). SET-PT is a two-step reaction involving the transfer of one electron followed by the donation of a proton. HAT involves the transfer of a hydrogen atom. SPLET involves the dissociation of a proton followed by the transfer of an electron from an ion [14,15,16,17].

Three mechanisms have been proposed for antioxidants to scavenge radicals such as DPPH, as shown in Figure 1. These are single-step hydrogen atom transfer (HAT), single electron transfer followed by proton transfer (SET-PT), and sequential proton loss electron transfer [14]. These mechanisms have varying eases of occurrence depending on the type of antioxidant, solvent, pH, and other conditions [11,15]. In addition, the ionization potential (IP), proton dissociation enthalpy (PDE), bond dissociation enthalpy (BDE), proton affinity (PA), and electron transfer enthalpy (ETE) are considered to play roles in these mechanisms, according to some studies [16,17]. Our study also uses these indicators to predict the values obtained in antioxidant tests through machine learning [18,19].

While there have been extensive structure–activity relationship (SAR) studies on food ingredients, most studies often only made comparisons based on differences in the substructures of compounds that share a common skeleton. For example, comparisons of 10 catechols and guaiacol [20]; derivatives of sinapic acid and caffeic acid [21]; ferulic acid, sinapic acid, and their reduced forms [22]; and phenol monomers and dimers [23] have been conducted. Generally, the higher the number of hydroxyl groups, the higher the DPPH radical scavenging capacity. However, carnosic acid with two phenolic hydroxyl groups has also been suggested to possess a higher antioxidant capacity than rosmarinic acid with four phenolic hydroxyl groups [24]. The possible reason for different trends being reported across several studies is that some of the reported trends are applicable only to a limited range of compounds. In addition, different laboratories use different experimental conditions, even though the measured values from the DPPH assay are greatly affected by factors such as the solvent used, incubation time, and temperature; there may also be differences in the manner in which the measurement results are processed. One study reported DPPH measurements for more than 100 compounds; however, it used a unique analytical method that required fewer experimental steps [25]. Overall, it is difficult to compare the results of measurements conducted in different laboratories because of their high dependency on the experimental conditions.

There is limited research on the use of SARs to determine the antioxidant capacity through the analysis of chemical components in contrast to the analysis of whole food components. This may partly be due to the scarcity of databases on foods. However, recently, the largest database on foods, FooDB [26], which contains tens of thousands of compounds, has been released. Naveja et al. estimated that 3228 compounds (13.5%) of the FooDB database are polyphenols [27]. We are interested in understanding which of these polyphenols and how many of them play a role in the antioxidant activity. It has been challenging to identify a method that accurately determines the antioxidant capacity; nevertheless, recent works have attempted to minimize errors in DPPH measurements among different laboratories [28]. In addition, since 2019, reagent companies have been commercially supplying a simple kit [29] based on this study. There are also several recent reports of component analysis using this DPPH kit [30,31,32,33,34,35,36].

In this study, to establish the DPPH assay as a low-cost screening tool, we conducted a versatile SAR investigation. We focused on a group of phenolic compounds and measured them under uniform conditions using the aforementioned kit. We calculated the Trolox equivalent antioxidant capacity (TEAC) according to the provided manual. The TEAC was calculated from the 50% inhibitory concentration (IC_50_). Our SAR study of the DPPH radical scavenging activity of a wide range of phenolic acid compounds used the largest dataset to date and may provide deeper insights based on the values obtained for the DPPH radical scavenging activity. In addition, we discuss the structural differences that may change these values by comparing our data with data from the hydrophilic-oxygen radical absorbance capacity (H-ORAC) method [37]. We also estimated whether the phenols listed in FooDB react with DPPH. We believe that through such an SAR investigation, we can deepen our understanding of antioxidants and their activity relationships and gain clarity on the general principles that underlie them.

## 2. Materials and Methods

### 2.1. Reagents and Synthesis

**Figure 2:**
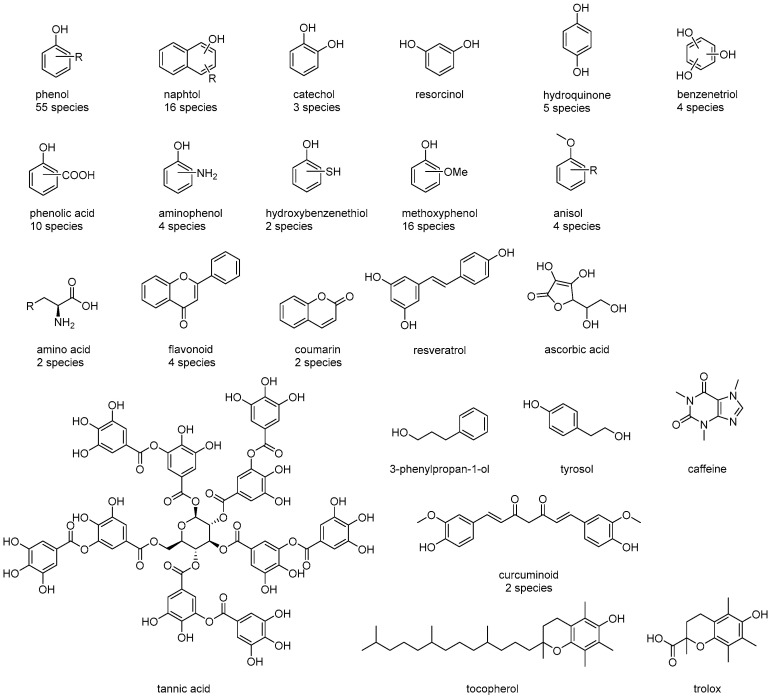
Examples of compounds and reference substances used in the assay. These compounds are commercially available. Other compounds are listed in the Supplementary Materials.

We purchased 142 reagents from WAKO (Tokyo, Japan), TCI (Tokyo, Japan), ALDRICH (Tokyo, Japan), Thermofisher (Fukuoka, Japan), JUNSEI (Kyoto, Japan), KANTO (Tokyo, Japan), MERCK (Tokyo, Japan), and Thermo Scientific (Fukuoka, Japan), focusing on known antioxidants in food ingredients and their derivatives (Figure 2). The reagents were used for the assay without purification. The reagent numbers and other information for each reagent are provided in the Supplementary Materials.

**Figure 3:**
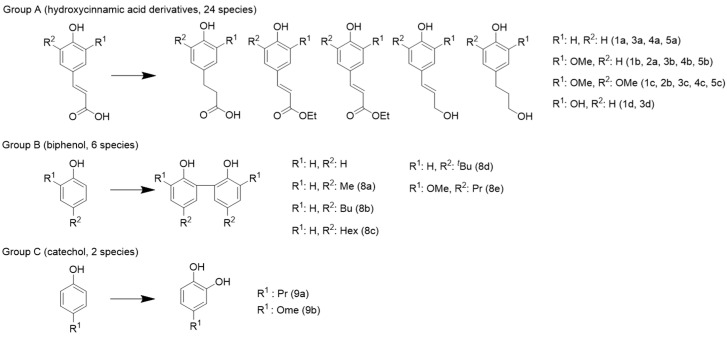
Substructures known to improve the antioxidant capacity that were synthesized in this study. Details are listed in the Supplementary Materials.

Twenty-seven compounds could not be directly purchased and were therefore synthesized. Representative compounds are shown in Figure 3. Ferulic acid, sinapic acid, and their reduced forms [22] (Group A); phenol monomers and dimers [23] (Group B); and catechol and 10 guaiacol species [20] (Group C) could be synthesized, as they have been studied individually in the literature. Details on the specific compounds synthesized and the employed methods are provided in the Supplementary Materials.

### 2.2. DPPH Radical Scavenging Activity Measurements

To evaluate the DPPH radical scavenging activities, the DPPH Antioxidant Assay Kit [29] of Dojin Kagaku Kenkyusho Co. was purchased from WAKO (Tokyo, Japan) and used as is, in accordance with its procedure manual. The absorbance was measured using Thermo Fisher Scientific’s ScanIt RE software for Varioskan Flash version 2.4. In preliminary experiments, sample solutions of 1, 10, 100, and 1000 μg/mL were reacted with specific amounts of the DPPH solution to calculate the optimal concentration range. The optimal concentration range is one with a concentration that eliminates 50% of the initial concentration of DPPH. The regression line was calculated from the DPPH scavenging rate when the sample solution was reacted with four concentration points in the optimal concentration range, and the IC_50_ was determined. To make corrections for the variation from test session to test session, the antioxidant activity in the DPPH assay was corrected using the IC_50_ of 6-hydroxy-2,5,7,8-tetramethyl-3,4-dihydrochromene-2-carboxylic acid (Trolox) measured for each test session. The TEAC was calculated according to Equation (1). The same reaction was performed in three wells for each concentration of each sample and blank, and the standard deviation was determined. All DPPH radical scavenging activities presented in this paper are the averages of these values. For samples with an IC_50_ greater than 1000 μg/mL, the IC_50_ was not subsequently measured in detail because the TEACs are expected to be lower.

(1) $$TEAC=\frac{IC_{50Trolox}}{IC_{50sample}}$$

### 2.3. Food Component Dataset

SMILES, a database with a linear notation of chemical structures, was downloaded from FooDB [26] in a batch in February 2023. In addition, the extraction of structures that are neutral molecules and have more than one aromatic hydroxy group was performed using RDKit [38]. This yielded 4240 compounds.

### 2.4. Calculations

For the compounds measured in this study (169 compounds), as well as those obtained from searching the literature (24 compounds) [30,31,32,33,34,35,36] and FooDB [26] (4240 compounds), we searched for stable conformations in three dimensions. Using RDKit [38], 1000 conformations were generated using the ETDKG method [39]; each structure was then optimized using the MMFF force field [40], and the most stable structure was used as the initial conformation for the semi-empirical quantum chemical calculation of neutral molecules. The extraction of aromatic OH was calculated using one of the RDKit methods, fr_Ar_OH.

For the 169 compounds measured, the heats of the formations of neutral, anion, radical, and radical cation states were determined using the Molecular Orbital Package (MOPAC2016) [41], a semi-empirical quantum chemical calculation program, with the PM7 [42] method. The IP, BDE, PDE, PA, and ETE were calculated using Equations (2) through (6), which are presented below. For the anions and radicals, the BDE and PA, which have the lowest calculated heats of formation in the molecule, were used. The values of H(e^−^) were quoted in a vacuum [43]. For the compounds extracted from FooDB (4240 species) and those whose DPPH radical scavenging activity was measured in the literature [30,31,32,33,34,35,36] (24 species), only the IP was calculated according to the same method. The details of the calculation method are described in the Supplementary Materials.

(2) $$BDE=H\left(ArO^{∙}\right)+H\left(H^{∙}\right)-H\left(ArOH\right)$$

(3) $$IP=H\left(ArOH^{+}\right)+H\left(e^{-}\right)-H\left(ArOH\right)$$

(4) $$PDE=H\left(ArO^{∙}\right)+H\left(H^{+}\right)-H\left(ArOH^{+}\right)$$

(5) $$PA=H\left(ArO^{-}\right)+H\left(H^{+}\right)-H\left(ArOH\right)$$

(6) $$ETE=H\left(ArO^{∙}\right)+H\left(e^{-}\right)-H\left(ArO^{-}\right)$$

## 3. Results and Discussion

### 3.1. Results of DPPH Measurements

Preliminary experiments were conducted on 169 compounds (including synthesized ones), 77 of which were found to have IC_50_ values of 1000 μg/mL or less. Therefore, further experiments were conducted on these 77 compounds to calculate their IC_50_ and TEAC values. The remaining 92 compounds were not examined in detail, because the values were expected to be smaller than 0.06 TEmol/mol. The examined compounds, those used as non-phenol, included aminoacids (L-phenylalanine, L-tryptophan), anisole derivatives (*p*-methylanisol, 2,3,4-trimethoxybenzaldehyde, (2-methoxy-4-prop-2-enylphenyl)acetate, 4-allylanisole), alcohols (3-phenylpropan-1-ol, caffeine, coumarin), (4-Prop-2-etnylphenyl)acetate, 4-allylanisole, 3-phenylpropan-1-ol, caffeine, coumarin, and flavanone featured TEACs of less than 0.06 TEmol/mol. All measured data and calculated values are shown in the Supplementary Materials. Although the TEACs (TEmol/mol) are mentioned and discussed in this paper, the Supplementary Materials also include the IC_50_ [mg/L], TEAC [gTE/g], and standard deviation of each measured value.

The highest TEAC in this study was 15.4 TEmol/mol for tannic acid. For many commercially available simple phenols, the range was 0 to 4.0 TEmol/mol. There have been several synthesis studies reporting increases in the activity of compounds. Our laboratory also performed these syntheses and replicated these results. The syntheses involved the reduction of adjacent olefins (Group A), coupling of phenols (Group B), and introduction of OH groups on the aromatic ring (Group C). A quantitative evaluation of these effects showed that the average increase in the TEAC was 0.1 TEmol/mol due to the coupling of phenols, 0.3 TEmol/mol due to the reduction of adjacent olefins, and 1 TEmol/mol due to the introduction of a hydroxy group at the *o*-position of the phenolics.

### 3.2. Relationships between the TEACs of DPPH and the BDE, IP, PDE, PA, and ETE

**Figure 4:**
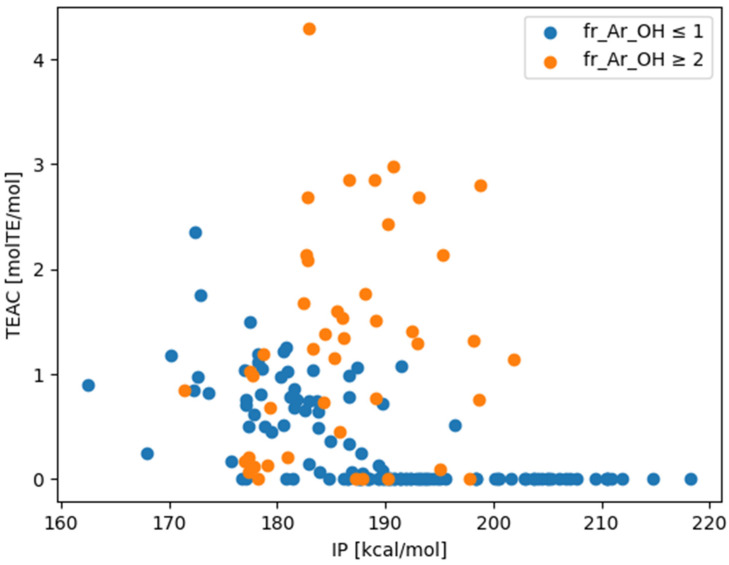
Relationship between the ionization potential (IP) calculated using MOPAC/PM7 and the Trolox equivalent antioxidant capacity (TEAC) values from the 2,2-diphenyl-1-picrylhydrazyl (DPPH) assay. Tannic acid with TEAC [molTE/mol] = 15.4 was excluded for visibility. This scatter plot was drawn using the matplotlib library.

Given that the TEACs of DPPH were measured using various functional groups, no strong macroscopic correlations related to the employed simple calculation method were observed. The calculated BDE, IP, PDE, PA, and ETE values, as well as the TEACs of DPPH, were compared. The IP was found to be the most important indicator determining the presence or absence of activity in the DPPH assay, at least under the present experimental conditions. The IP is the reaction enthalpy of the first reaction in SET [14]. The overall trend was that more compounds with the same IP were more active when the aromatic attribute had two hydroxy groups than when it had one (Table 1). The measured TEAC and IP values are plotted on the vertical and horizontal axes, respectively, in Figure 4, and the datapoints are color-coded according to the number of phenolic hydroxy groups. Among the compounds measured, little activity was observed in compounds with IPs above 200 kcal/mol.

### 3.3. Comparison with the Literature Data

**Figure 5:**
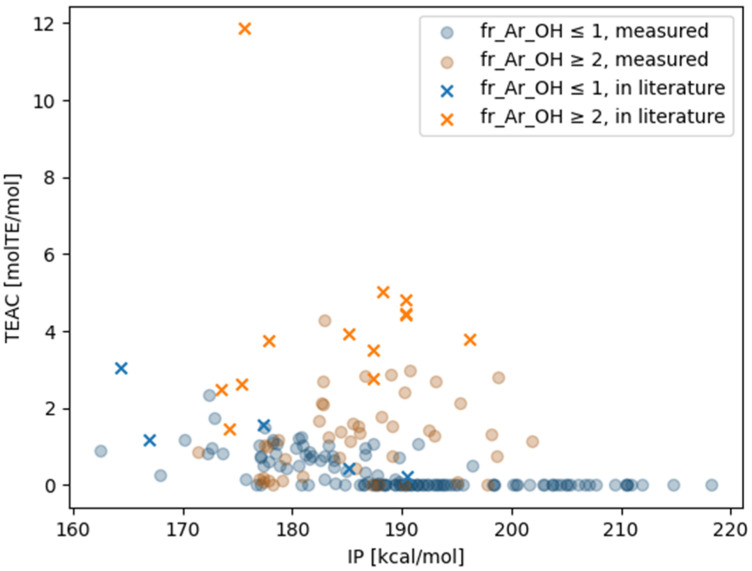
Comparison of IP and TEAC [molTE/mol] values in the literature measured using the same kit with the corresponding values of the measured compounds obtained in this study. Tannic acid with TEAC [molTE/mol] = 15.4 was excluded for visibility. “fr_Ar_OH” is the number of aromatic hydroxy groups; “measured” denotes values measured in our laboratory; “in literature” is the value measured with the same kit and reported elsewhere. All values measured in our laboratory are the averages of triplicate measurements. This scatter plot was drawn using the matplotlib library.

Comparisons were made with data obtained using the same experimental kit as in the current study [30,31,32,33,34,35,36]. There was a general agreement of values for eight species between the literature data [30,31,32] and measured data (Table 2). In addition, all 24 data types that were not included in the measured data but included in the literature data had TEACs [molTE/mol] greater than 0.2. IP calculations were performed for these data types, and the largest value was found to be 195.6 kcal/mol (Figure 5). The maximum IP value was 176.6 kcal/mol among compounds with a single aromatic hydroxy group. These were IP values that were assumed to correspond to a TEAC of 0.2 or higher, considering Figure 4.

### 3.4. SAR of DPPH

Table 3 provides information on the individual substituents. The effects of the substituents are discussed individually below. The trends of important substituents, such as the number of electron-donating groups, electron-withdrawing groups, and aromatic hydroxy groups, which have been confirmed in the past, are examined in detail.

#### 3.4.1. Electron-Donating and Electron-Withdrawing Groups

A comparison of compounds with one benzene ring and one aromatic hydroxy group with and without substituents revealed a general tendency for TEACs to be higher for compounds with electron-donating groups than for compounds with electron-withdrawing groups. For substituents at the *p*-position, the order of activity was as follows: methoxy (entry 23) > 1000 μg/mL > H, nitrile, carbonyl, ketone, nitro, alkyl. Similarly, for substituents at the *o*-position, the order of activity was as follows: methoxy (entry 19) > 1000 μg/mL > H, carbonyl, carboxy, alkyl. For compounds with one methoxy group at the *o*-position and different substituents at the *p*-position, the TEACs were in the following order: alkyl > H > carbonyl (entries 22, 19, 24). For the methoxy group at the 2,6-position, the substituents at the *p*-position were also in the order of alkyl > H > carbonyl. In addition, except for compounds with two or more hydroxy, mercapto, or amino groups substituted in one aromatic ring, the trend of electron-donating group > H > electron-withdrawing group was also observed for compounds with only one other substituent (entry 25 > entry 28).

#### 3.4.2. Number and Position of Aromatic Attribute Hydroxy Groups

To discuss the effect of the number of hydroxy groups on activity, a comparison was made among compounds with one or more hydroxy groups substituted on a single aromatic ring. Compounds with two or more hydroxy groups in the same orientation exhibited higher TEACs than those with only one. The hydroxy group is an electron-donating group, which may be the first reason for facilitating the reaction. However, hydroxylated compounds exhibited higher TEACs than compounds with a methoxy group (considered to be a stronger electron donor) in the same position. For hydroquinone (entry 16), the reaction of phenoxy radicals regenerated one hydroxy group, while the other became a quinone. The formed quinone regenerated hydroxy groups under solvent attack in alcohols [41]. This is the possible reason why compounds with the structures of catechol (entry 12) and hydroquinone (entry 16) exhibited very large TEACs. Notably, the TEACs of compounds with three or more hydroxy groups on the same benzene ring were not necessarily greater than those of compounds with two hydroxy groups (entries 16 and 12 vs. entries 4, 5, and 7). The attack of alcoholic solvents on quinone is promoted by the presence of electron-withdrawing groups [41], and the presence of three or more hydroxy groups may inhibit this reaction, as these groups are strong electron donors. In fact, hydroquinone (entry 16) with electron-withdrawing bromine or carboxy groups (entries 13, 14, 27) showed a higher TEAC than hydroquinone (entry 16). Moreover, 4-[bis(4-hydroxyphenyl)methyl]phenol has three aromatic hydroxy groups in the molecule as a whole but has a TEAC of less than 0.2, because each of the three different aromatics has a hydroxy group, the TEACs of which are lower than those in entries 4, 5, 7.

#### 3.4.3. Phenols with Amino and Mercapto Groups

Anilines and thiols, similar to phenol, have been reported to scavenge DPPH radicals by releasing hydrogen atoms [42,44,45]. Amino and mercapto groups are stronger electron donors than the hydroxy group, and the corresponding compounds have lower calculated IPs than phenol (entry 26), resorcinol (entry 29), and hydroquinone (entry 16). In particular, 2-aminophenol (entry 2), together with catechol (entry 12), shows a particularly high TEAC. This suggests that a reaction similar to the regeneration of hydroxy groups by alcoholic solvent attack after the formation of quinone from catechol (entry 12) and hydroquinone (entry 16) may occur with the amino groups.

#### 3.4.4. Effect of Dimerization on DPPH Activity

Despite the presence of a hydroxy group in each of the two different aromatics, the TEACs tended to improve for ortho-ortho-coupling phenols. The TEACs of the dimers were calculated for four of the entries (8–11). For three of the entries without ortho substituents, the TEAC of the monomer was less than 0.05 [molTE/mol], and the ortho-ortho coupling of phenol increased the activity more than two-fold. A synergistic effect was observed. An exception was 2-methoxy-4-propylphenol (entries 9, 20), which exhibited higher TEACs before coupling and decreased TEACs after coupling. The TEAC of tannic acid (entry 30), which is a five-added structure of gallic acid (entry 6), was also higher than that of its unit structure, gallic acid. Additive effects due to the sum of partial structures were also identified.

### 3.5. Comparison of H-ORAC and DPPH Assay Results

We compared the TEAC activity of DPPH measured in the present study with the previously determined H-ORAC activity [37]. Takebayashi et al. evaluated the total hydrophilic antioxidant content of 23 vegetables and 13 fruits commonly consumed in Japan using the H-ORAC method. They reported that the H-ORAC values were strongly positively correlated with the polyphenol content (*r* = 0.956) and were 1.0–18.2 times higher than the TEACs evaluated using the DPPH assay [8]. The overall tendency of obtaining higher values with the H-ORAC method was also confirmed when comparing the ~60 phenol components available for comparison in this study.

Three factors were identified in this study as influencing the respective values for the H-ORAC and DPPH assays, namely with differences in (i) measurement methods, (ii) mechanisms, and (iii) the reactivity of the reference material (Trolox). The first factor, i.e., the difference between the two methods, is that the H-ORAC assay uses a competitive reaction between active radical species generated in the system and antioxidants and fluorescent substances. Hence, the reaction rate is an important factor in determining activity with this method. Consequently, the reaction barrier is considered to have a significant effect on activity. On the other hand, in the case of the DPPH assay, the value obtained is thermodynamic when calculated through IC_50_ after 30 min of reduction, as in the present experiment [3]. The second factor, i.e., the difference in mechanisms, is that the reaction mechanisms of the two assay methods are different, with H-ORAC being more influenced by HAT and DPPH being more influenced by SET-PT. Trolox has a methyl group at the *o*-position, which contributes to its activity in SET-PT; however, its steric effect lowers the reaction rate in HAT. The third factor is that the other compounds have TEACs higher or lower than 1 [molTE/mol], depending on the nature of the reference compound used.

**Figure 6:**
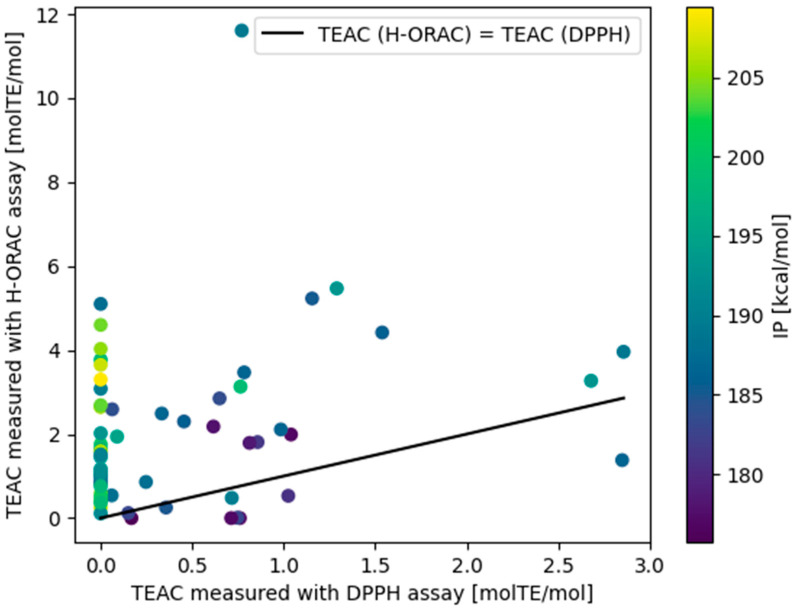
Comparison of TEACs measured in the DPPH (the present study) and hydrophilic oxygen radical absorbance capacity (H-ORAC; reported in ref. [24]) assays. This scatter plot was drawn using the matplotlib library. Each datapoint is colored according to the size of the IP, with the corresponding color scale shown in the bar on the right. The straight line corresponds to (TEAC in the ORAC test) = (TEAC in the DPPH test) and serves as a visual guide to facilitate the comparison between the two classes of TEACs.

The DPPH assay showed that compounds with a high IP had low activity. On the other hand, according to the H-ORAC assay, which has been noted to be influenced by HAT, compounds with a high IP that did not show activity in the DPPH assay often showed IP values of ~5 in the H-ORAC assay (Figure 6). In the H-ORAC assay, the tert-butyl group at the *o*-position, which greatly affects the reaction rate of HAT, significantly lowered the activity, whereas in the DPPH assay, the effect was small (Table 4, entry 3). Trolox, which was used as a reference substance in both tests, has a methyl group adjacent to the aromatic OH group, and due to its steric effect, it showed relatively low activity in the H-ORAC assay and relatively high activity in the DPPH assay by virtue of the electron-donating nature of the methyl group (Table 4, entries 0–2).

### 3.6. Comparison with FooDB Compounds

**Figure 7:**
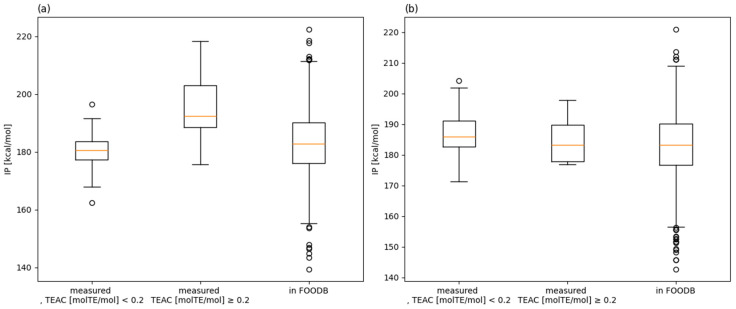
Comparison of the distribution of the IP for the measured compounds and FooDB with a TEAC of 0.2 or less and a TEAC of 0.2 or greater. Compounds with less than (**a**) one aromatic hydroxy group and (**b**) those with more than two aromatic hydroxy groups were analyzed separately. These box-and-whisker diagrams were drawn using the matplotlib library. The box extends from the first quartile to the third quartile of the data, with a line at the median. The whiskers extend from the box to the farthest datapoint lying within 1.5× the interquartile range from the box. Flier points are those past the end of the whiskers.

Finally, the IP of the food group was calculated and measured using the DPPH assay, and its distribution was compared to that of the compound groups with a TEAC greater than 0.2 [molTE/mol] and a TEAC less than 0.2 [molTE/mol] (Figure 7). Since the trends differed depending on the position and number of aromatic hydroxyl groups, they were analyzed separately. Of the compounds for which calculations were completed, 1503 had one aromatic hydroxyl group in FooDB, and 2737 had two or more. The details of these results are provided in the Supplementary Materials. For the 169 compounds we measured, the IP above a certain level was estimated to be less than 0.2 mol/mol for the TEAC; for the phenols listed in FooDB, many IPs were estimated to be greater than or equal to 0.2 molTE/mol for the TEAC. This is because many phenols registered with FooDB feature electron-donating (e.g., methoxy) groups on their aromatic rings. To our best knowledge, no such comprehensive large-scale predictions have ever been made. We believe that further structure–activity correlations will facilitate the calculation of predictions prior to measurement.

## 4. Conclusions

DPPH measurements used to determine TEACs were performed on 169 compounds that are components of foods and have similar structures. The SARs of the measured compounds were investigated and summarized. The obtained dataset provides insights into the effects of the combination of multiple factors and their relationship with each other, which has only been partially discussed thus far by measuring a large number of compounds under the same experimental conditions. We found a weak correlation between the IP and TEAC under the present experimental conditions, suggesting that many of the measured compounds reacted via SET-PT. Using the H-ORAC and DPPH assays, we were able to compare measurements for ~60 compounds under uniform experimental conditions for each method. We observed several trends in the measured TEACs from the H-ORAC and DPPH assays. In the large dataset obtained in this study, no strong macroscopic correlations related to the DPPH assay-derived values were observed; nevertheless, different TEAC trends were observed for each functional group. We will continue to conduct experiments to expand the dataset; nevertheless, our present database will be useful for creating new benchmarks and large-scale in silico tools for molecular design, based on our analysis of FooDB compounds. Our laboratory is also exploring the creation of predictive models in silico that consider not only the IP, but also steric, substituent, and electronic factors, as well as the effects of individual substituents. We are also attempting to calculate the velocity data as well as thermodynamic values for these compounds.

## Figures and Tables

**Table 1:** Calculated parameters for the aromatic hydroxy groups and the TEACs measured using the DPPH assay.

	Mean (fr_Ar_OH ≤ 1)	Std (fr_Ar_OH ≤ 1)	Mean (fr_Ar_OH ≥ 2)	Std (fr_Ar_OH ≥ 2)
IP [kcal/mol]	189.6	11.0	186.6	7.5
BDE [kcal/mol]	77.0	5.5	76.3	4.5
PA [kcal/mol]	272.9	10.8	266.5	7.4
PDE [kcal/mol]	143.4	9.4	145.8	7.1
ETE [kcal/mol]	60.2	12.2	65.9	9.2
TEAC [molTE/mol]	0.31	0.48	1.25	1.05

Activities and calculation results when the number of aromatic hydroxy groups is greater than 2 and less than 1. IC_50_ values exceeding 1000 μg/mL were calculated as TEAC [molTE/mol] = 0. The TEACs [molTE/mol] of all compounds with IC_50_ values exceeding 1000 μg/mL were less than 0.2, and even taking into account that the TEAC was set to 0, there were differences in the average TEACs depending on the number of hydroxy groups. All measurements in our laboratory were repeated three times, and the results are reported as the corresponding means.

**Table 2:** Comparison of TEACs for DPPH tested with the same kit as that used in this study, for the same compounds.

Compound	TEAC [molTE/mol] (Measured)	Standard Deviation (Measured)	TEAC [molTE/mol] (Literature)	Ref.
L-ascorbic acid	1.08	0.04	0.91	[32]
Catechin	2.43	0.07	3.08	[30]
Catechin	2.43	0.07	2.32	[31]
Quercetin	4.29	0.04	3.96	[30]
Sesamol	0.98	0.05	1.04	[30]
Ferulic acid	0.78	0.05	0.80	[30]
Gallic acid	2.81	0.07	3.07	[30]
Morin	0.99	0.12	1.33	[30]
D-Alpha-tocopherol	0.90	0.12	1.90	[30]

All measurements in our laboratory were repeated three times, and the results are presented as the corresponding means.

**Table 3:** Changes in the TEAC when each structure is substituted from X^1^ to X^5^.

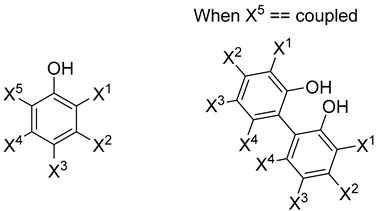									
Entry	IUPAC Name (Common Name)	Category	X^1^	X^2^	X^3^	X^4^	X^5^	TEAC [molTE/mol]	Standard Deviation
0	6-hydroxy-2,5,7,8-tetramethylchromane-2-carboxylic acid (Trolox)	Trolox	-	-	-	-	-	1.0	-
1	3-aminophenol	aminophenol	H	SH	H	H	H	0.76	0.06
2	2-aminophenol	aminophenol	NH_2_	H	H	H	H	2.36	0.01
3	4-aminophenol	aminophenol	H	NH_2_	H	H	H	0.76	0.02
4	benzene-1,2,4-triol	benzenetriol	OH	H	OH	H	H	0.46	0.06
5	benzene-1,2,3-triol	benzenetriol	OH	OH	H	H	H	2.85	0.12
6	3,4,5-trihydroxybenzoic acid (gallic acid)	benzenetriol	OH	H	COOH	H	OH	2.81	0.07
7	benzene-1,3,5-triol	benzenetriol	H	OH	H	OH	H	0.76	0.03
8	2-(2-hydroxy-5-methylphenyl)-4-methylphenol	biphenol	H	H	Me	H	coupled	0.21	0.02
9	2-(2-hydroxy-3-methoxy-5-propylphenyl)-6-methoxy-4-propylphenol	biphenol	OMe	H	propyl	H	coupled	0.85	0.13
10	4-butyl-2-(5-butyl-2-hydroxyphenyl)phenol	biphenol	H	H	butyl	H	coupled	0.12	-
11	4-hexyl-2-(5-hexyl-2-hydroxyphenyl)phenol	biphenol	H	H	hexyl	H	coupled	0.14	0.04
12	benzene-1,2-diol (catechol)	catechol	OH	H	H	H	H	2.86	0.03
13	2-chlorobenzene-1,4-diol	hydroquinone	Cl	H	OH	H	H	1.41	0.04
14	2-bromobenzene-1,4-diol	hydroquinone	Br	H	OH	Br	H	2.14	0.12
15	2-methylbenzene-1,4-diol	hydroquinone	Me	H	OH	H	H	1.15	0.06
16	hydroquinone	hydroquinone	H	H	OH	H	H	0.77	0.02
17	2-mercaptophenol	hydroxybenzenethiol	SH	H	H	H	H	0.77	0.27
18	4-mercaptophenol	hydroxybenzenethiol	H	H	SH	H	H	0.74	0.04
19	2-methoxyphenol	methoxyphenol	OMe	H	H	H	H	0.33	0.03
20	2-methoxy-4-propylphenol	methoxyphenol	OMe	H	propyl	H	H	1.25	0.08
21	2,6-dimethoxyphenol	methoxyphenol	OMe	H	H	H	OMe	0.61	0.05
22	2-methoxy-4-methylphenol	methoxyphenol	OMe	H	Me	H	H	0.81	0.06
23	4-methoxyphenol	methoxyphenol	H	H	OMe	H	H	0.65	0.06
24	4-hydroxy-3-methoxybenzaldehyde (vanillin)	methoxyphenol	OMe	H	CHO	H	H	0.33	0.00
25	2,6-dimethylphenol	phenol	Me	H	H	H	Me	0.71	0.06
26	phenol	phenol	H	H	H	H	H	<0.03	0.00
27	2-caroboxybenzene-1,4-diol	phenolic acid	COOH	H	OH	H	H	1.29	0.16
28	2-hydroxy-3-methylbenzoic acid (3-methylsalicylic acid)	phenolic acid	Me	H	H	H	COOH	<0.05	-
29	benzene-1,3-diol (resorcinol)	resorcinol	H	OH	H	H	H	0.09	0.06
30	tannic acid	tannic acid	-	-	-	-	-	15.4	1.30

All measurements in our laboratory were repeated three times, and the results are reported as the corresponding means. IC_50_ [mol/L] and TEAC [mgTE/mg] values and their standard deviations are shown in the Supplementary Materials.

**Table 4:** Differences in TEACs between the H-ORAC and DPPH assays for specific compounds.

Entry	Compound	TEAC (H-ORAC) [molTE/mol]	Standard Deviation	TEAC (DPPH) [molTE/mol]	Standard Deviation
0	Trolox	1.0	-	1.0	-
1	Phenol	1.76	0.160	<0.03	-
2	2,6-dimethylphenol	0.48	0.068	0.72	0.06
3	2,6-di-tert-butylphenol	<0.01	-	0.75	0.20

All measurements in our laboratory were repeated three times, and the results are presented as the corresponding means.

## Data Availability

The data presented in this study are available on request from the corresponding author.

## Supplementary Materials

The following supporting information can be downloaded at https://www.mdpi.com/article/10.3390/antiox13030309/s1: SI.docx (Figure S1: Scatter plot matrix of calculated values of compounds measured in the lab through MOPAC2016 (PM7), Figure S2: Relationship between the calculated values and TEACs [molTE/mol] of compounds measured in the lab, Figure S3: Box-and-whisker diagram of TEAC values in the DPPH test for post-synthesized (synthesized) and pre-synthesized (reactants) compounds, Table S1: used key word for MOPAC2016 (PM7) calculation, Table S2: IC_50_ and TEAC of catechin, Table S3: IC_50_ of Trolox, Table S4: Correlation coefficients for IP, BDE, PA, PDE, and ETE values calculated in MOPAC2016 (PM7), Table S5: Means and standard deviations for active (TEAC [molTE/mol] ≥ 0.2) and inactive (TEAC [molTE/mol] < 0.2) compounds); TEAC_measured_with_DPPH_test_in_the_lab.xlsx: DPPH measured values (IC_50_ [mg/L], TEAC [molTE/mol], etc.) for 169 compounds; TEAC_measured_with_the_same_kit_reported_in_literatures.xlsx; measured_H_ORAC_to_compare_with_DPPH.csv; calculation_result_of_phenols_in_FOODB.csv; SI(Synthesis).docx: Compound synthesis method [46,47,48,49,50,51,52] and the ^1^H-NMR spectra part of synthesized compounds.
