# Supplementary material for: DPPH Measurements and Structure—Activity Relationship Studies on the Antioxidant Capacity of Phenols
Source: Antioxidants (Basel). 2024 Mar 1;13(3):309. doi: 10.3390/antiox13030309 (PMC10967577; doi:10.3390/antiox13030309)
Supplement: Supplementary file 1 [file antioxidants-13-00309-s001.zip › SI(Synthesis).pdf]

Compound synthesis method

1. Synthesis of Sinapinic acid **0a** compounds

*p*-coumaric acid, Ferulic acid, and Caffeic acid were commercially available and used without purification; Sinapinic acid was synthesized according to Wang Xiaojing et al [46] to obtain 2.56 g in 98% yield. The compound was checked for consistency with existing data [47].

2. Synthesis of **1a, 1b, 1c, 1d** compounds

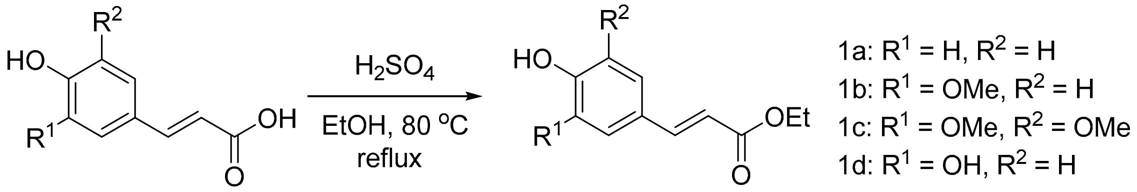


**1a** (1.71 g, 97% yield), **1b** (87% yield), **1c** (82% yield), and **1d** (80% yield) were synthesized from p-coumaric acid, Ferulic acid, Caffeic acid, and Sinapinic acid, following the method of Chao Wang et al. [48] yield), **1b** (87% yield), **1c** (82% yield), and **1d** (80% yield) were synthesized from the compound.

3. Synthesis of **2a, 2b, 3a, 3c, 3d** compounds

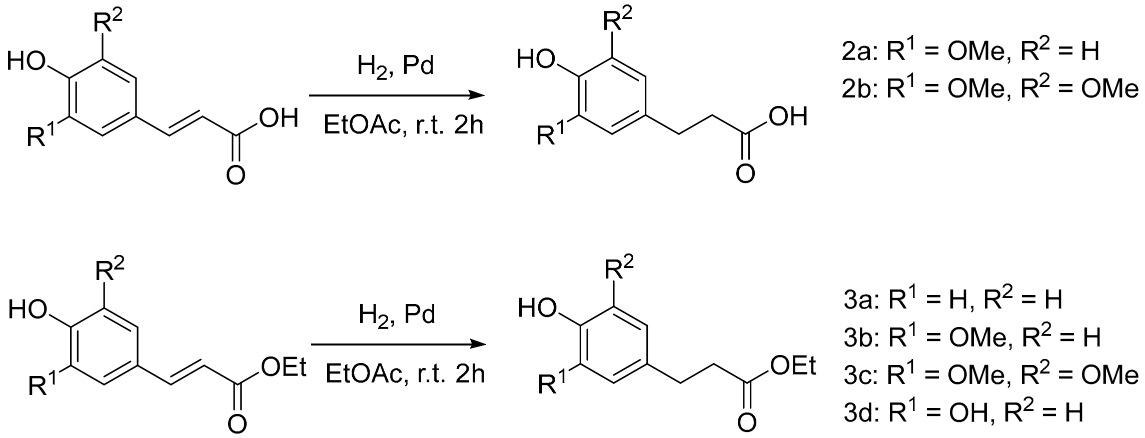


**2a**, **2b**, and **3a**~**3d** were synthesized from **1a**~**1d**, following the method of Liu, Jitian et al [49] to obtain products **3a** (0.27 g, 82% yield), **2a** (85% yield), **2b** (80% yield), **3b** (76% yield), **3c** (75% yield), and **3d** (83% yield).

4. Synthesis of **4a, 4b, 4c, 5a, 5b, 5c** compounds

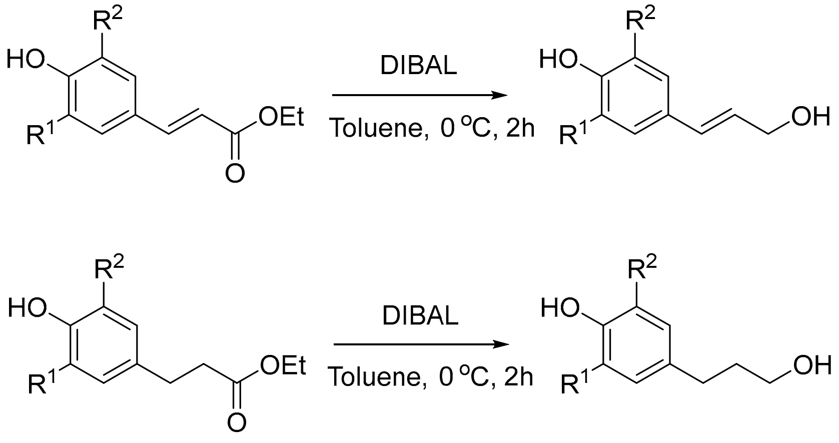


4a: $R^1$ = H, $R^2$ = H
4b: $R^1$ = OMe, $R^2$ = H
4c: $R^1$ = OMe, $R^2$ = OMe

5a: $R^1$ = H, $R^2$ = H
5b: $R^1$ = OMe, $R^2$ = H
5c: $R^1$ = OMe, $R^2$ = OMe

**4a**~**4c** and **5a**~**5c** were synthesized from **1a**~**1d** and **3a**~**3d**, respectively, following the method of Yue, Fengxia et al [50] to obtain **4a** (0.16 g, 44% yield), **4b** (52% yield), **4c** (55% yield), **5a** (50% yield ), **5b** (43% yield), and **5c** (52% yield) were obtained.

5. Synthesis of **6a**, **6b** compounds

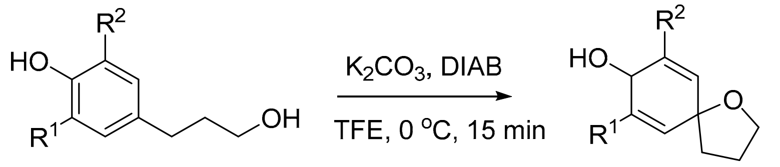


6a: $R^1$ = OMe, $R^2$ = H
6b: $R^1$ = OMe, $R^2$ = OMe

**6a** and **6b** were synthesized from **5b** and **5c** following the method of Li, Jiayin et al [51] to obtain **6a** (70 mg, 40% yield) and **6b** (34% yield).

6. Synthesis of **7b** compound

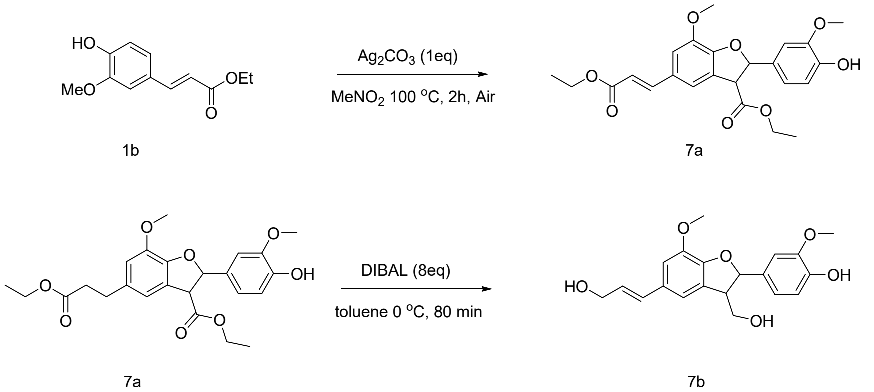


To begin with, **7a** was synthesized from **1b** by dimerization reaction. The procedure is described below. **1b** (101.6 mg, 0.45 mmol) and $MeNO_2$ (4.5 mL) were added to a pressure-resistant vessel under air. To this solution was added $Ag_2CO_3$ (126.3 mg, 0.45 mmol) and the solution was stirred in an oil bath at 100 °C for 2 h. The solid was removed by suction

filtration using Celite and the resulting solution was concentrated by rotary evaporator to give the crude product. Isolation was performed by thin layer chromatography ($SiO_2$, Ethyl acetate:Hexane=1:1) to obtain **7a** (36.2 mg, 35.6% yield).The resulting **7a** was then reduced using DIBAL in toluene for 80 min to give **7b**.

7. Synthesis of **8a, 8b, 8c, 8d, 8e** compounds

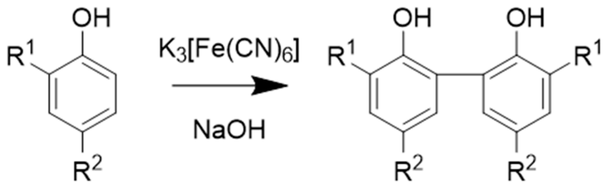


8b: $R^1$: H, $R^2$: Bu

8c: $R^1$: H, $R^2$: Hex

8d: $R^1$: H, $R^2$: $^t$Bu

8e: $R^1$: OMe, $R^2$: Pr

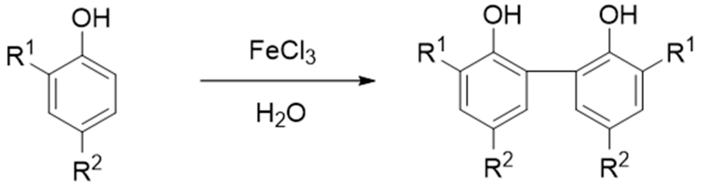


8a: $R^1$ = H, $R^2$ = Me

The following procedure was used to synthesize 8a: 0.9 mmol of the raw material and 1.8 mL of pure water were put into the reaction vessel and stirring was started. Anhydrous iron(III) chloride 0.6 mmol was added directly to the system and allowed to react for 24 hours at room temperature. After the reaction, the organic layer was extracted with chloroform. The organic layer was dehydrated with sodium sulfate and the solvent was removed under reduced pressure to give the crude product. Isolation was performed by thin layer chromatography ($SiO_2$, Ethyl acetate:Hexane) to obtain **8a**.

**8b, c, d**, and **e** were synthesized by the following procedure. 0.6 mmol of the raw material and 1.8 mL of 0.5 mol/L sodium hydroxide solution were added to the reaction vessel and stirring was started. 0.6 mmol of potassium ferricyanide was directly fed into the system and allowed to react for 24 hours at room temperature. After the reaction, the reaction solution was neutralized with hydrochloric acid and the organic layer was extracted with chloroform. The organic layer was dehydrated with sodium sulfate and the solvent was removed under reduced pressure to give the crude product. Isolation was performed by thin layer chromatography ($SiO_2$, Ethyl acetate:Hexane) to obtain **8b, c, d**, and **e**.

8. Synthesis of **9a** and **9b** compounds

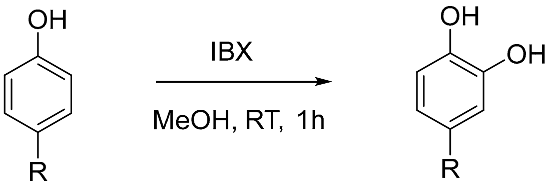


9a: R = Pr
9b: R = OMe

**9a** (26.5 mg, 34.8% yield) and **9b** (10.5 mg, 15.0% yield) were synthesized from *p*-propylphenol and *p*-methoxyphenol following the method of Roberta Bernini et al [52].

## Reference


46. Wang, X.; Feng, J.; Bai, Y.; Zhang, Q.; Yin, Y. Synthesis, Properties, and Applications of Hollow Micro-/Nanostructures. Chem. Rev. 2016, 11, 10983-11060. https://doi.org/10.1021/acs.chemrev.5b00731.
47. Salum, ML.; Robles, CJ.; Erra-Balsells, R. Photoisomerization of ionic liquid ammonium cinnamates: One-pot synthesis-isolation of Z-cinnamic acids. Org. Lett. 2010, 12, 4808-4811. https://doi.org/10.1021/ol1019508.
48. Wang, C.; Qian, C.; Roman, M.; Glasser, W. G.;Esker, A. R. Surface-initiated dehydrogenative polymerization of monolignols: A quartz crystal microbalance with dissipation monitoring and atomic force microscopy study. Biomacromolecules. 2013, 14, 3964-3972. https://doi.org/10.1021/bm401084h.
49. Liu, J.; Ye, W.; Wang, S.; Zheng, J.; Tang, W.; Li, X. Synthesis of Lactams via Ir-Catalyzed C–H Amidation Involving Ir-Nitrene Intermediates. J. Org. Chem.2020, 85, 4430-4440. https://doi.org/10.1021/acs.joc.0c00157.
50. Yue, F.; Lu, F.; Regner, M.; Sun, R.; Ralph, J. Lignin-derived thioacidolysis dimers: reevaluation, new products, authentication, and quantification. ChemSusChem, 2017, 10.5, 830-835. https://doi.org/10.1002/cssc.201700101.
51. Li, J.; Liang, J. L.; Chan, P. W. H.; Che, C. M. Aziridination of alkenes with N-substituted hydrazines mediated by iodobenzene diacetate. Tetrahedron lett., 2004, 45, 2685–2688. https://doi.org/10.1016/j.tetlet.2004.01.127.
52. Bernini, R.; Crisante, F.; Barontini, M.; Tofani, D.; Balducci, V.; Gambacorta, A. Synthesis and Structure/Antioxidant Activity Relationship of Novel Catecholic Antioxidant Structural Analogues to Hydroxytyrosol and Its Lipophilic Esters: J. Agric. Food Chem., 2012, 60, 30, 7408-7416. https://doi.org/10.1021/jf301131a.

$^{1}$H-NMR spectra part of synthesized compounds

Compound **1a**

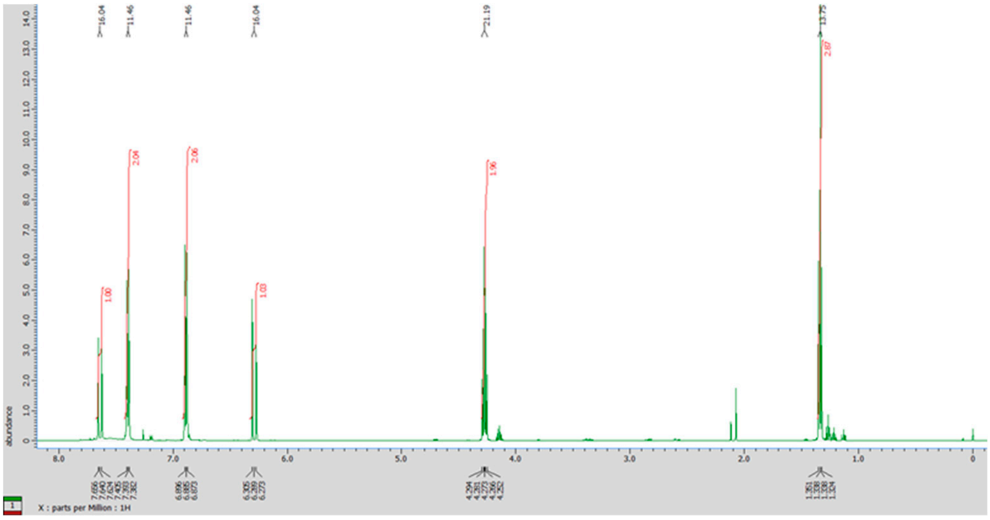

$^{1}$H-NMR (500 MHz, CHLOROFORM-D) δ 7.64 (d, $J$ = 16.0 Hz, 1H), 7.39 (d, $J$ = 11.5 Hz, 2H), 6.88 (d, $J$ = 11.5 Hz, 2H), 6.29 (d, $J$ = 16.0 Hz, 1H), 4.27 (q, $J$ = 7.1 Hz, 2H), 1.34 (t, $J$ = 6.9 Hz, 3H)

Compound **1b**

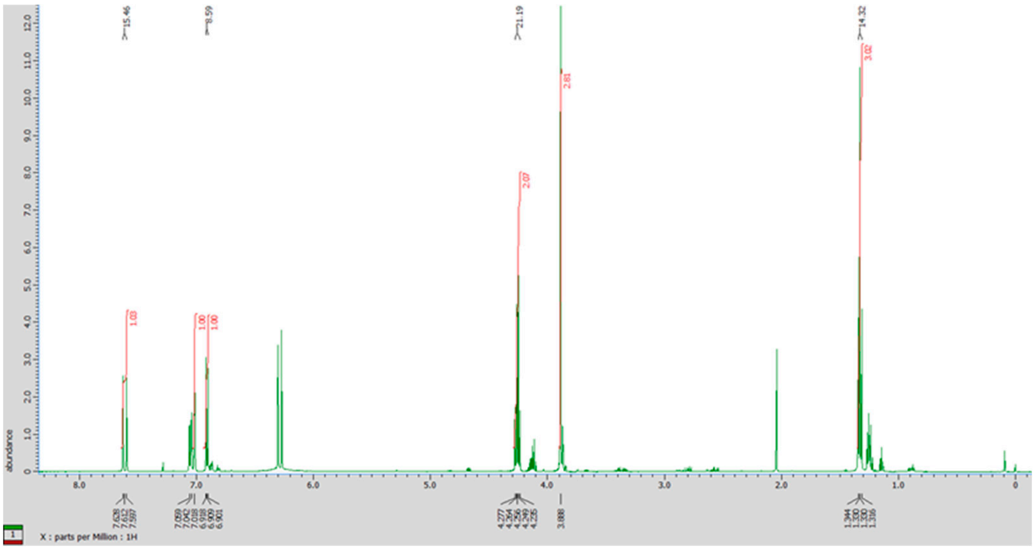

$^{1}$H-NMR (500 MHz, CHLOROFORM-D) δ 7.61 (d, $J$ = 15.5 Hz, 1H), 7.02 (s, 1H), 6.91 (d, $J$ = 8.6 Hz, 1H), 4.26 (q, $J$ = 7.1 Hz, 2H), 3.89 (s, 3H), 1.33 (t, $J$ = 7.2 Hz, 3H)

Compound **1c**

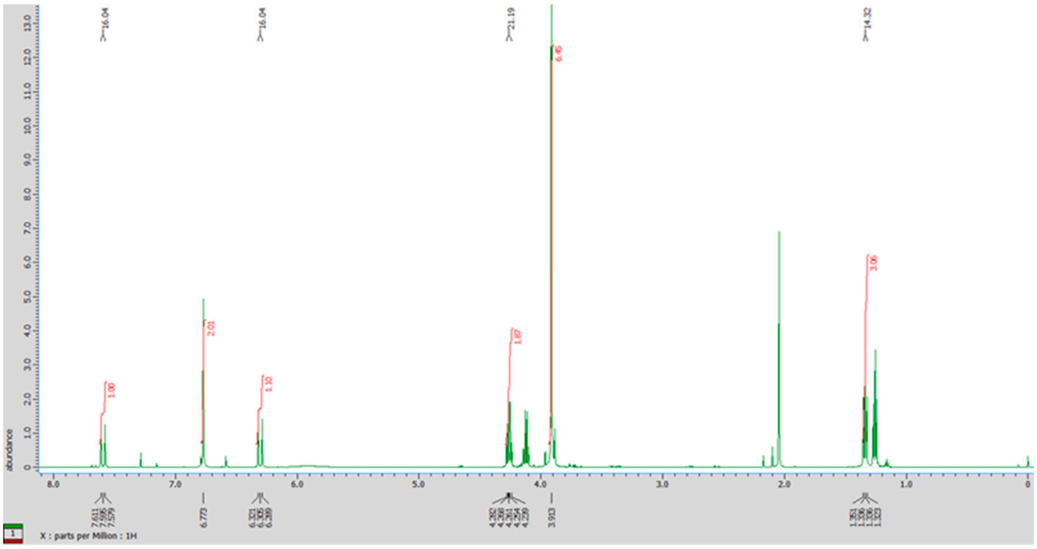

$^{1}$H-NMR (500 MHz, CHLOROFORM-D) δ 7.59 (d, *J* = 16.0 Hz, 1H), 6.75 (s, 2H), 6.30 (d, *J* = 16.0 Hz, 1H), 4.26 (dd, *J* = 14.9, 6.3 Hz, 2H), 3.89 (s, 6H), 1.33 (t, *J* = 7.2 Hz, 3H)

Compound **1d**

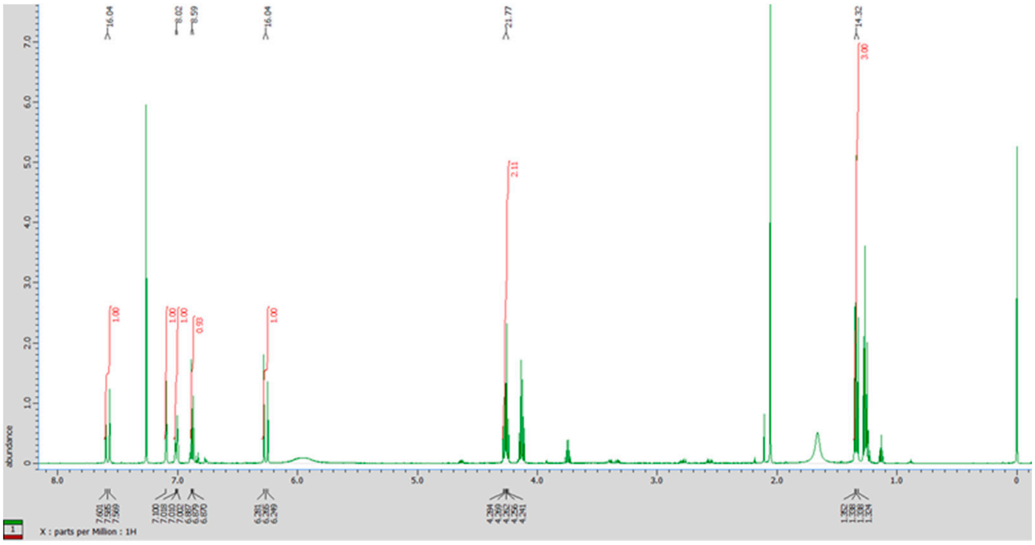

$^{1}$H-NMR (500 MHz, CHLOROFORM-D) δ 7.58 (d, *J* = 16.0 Hz, 1H), 7.10 (s, 1H), 7.01 (d, *J* = 8.0 Hz, 1H), 6.88 (d, *J* = 8.6 Hz, 1H), 6.27 (d, *J* = 16.0 Hz, 1H), 4.26 (q, *J* = 7.3 Hz, 2H), 1.34 (t, *J* = 7.2 Hz, 3H)

Compound **2a**

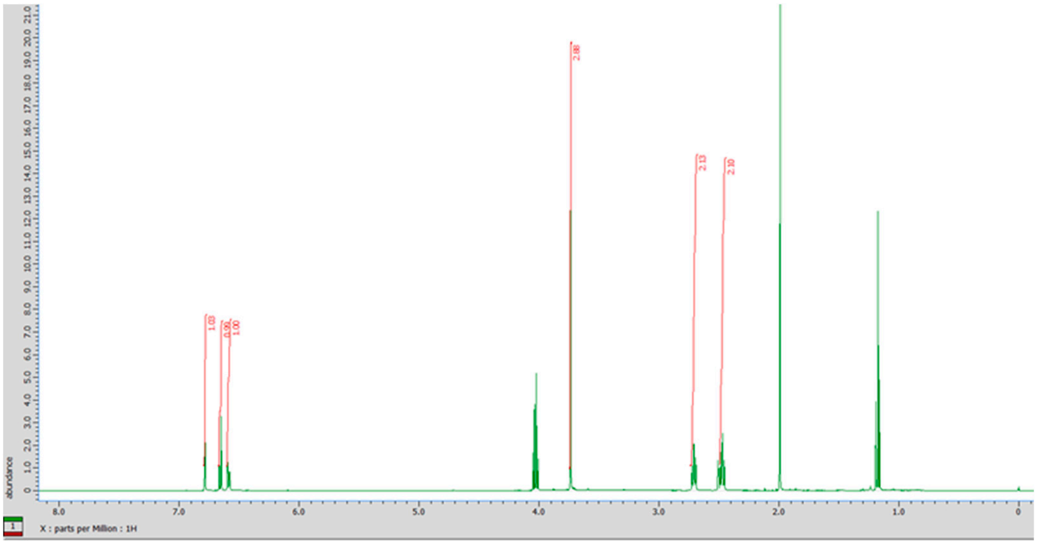


$^{1}$H-NMR (500 MHz, DMSO-D6) δ 6.78 (d, $J$ = 1.7 Hz, 1H), 6.66 (d, $J$ = 8.0 Hz, 1H), 6.59 (d, $J$ = 8.0 Hz, 1H), 3.74 (s, 3H), 2.71 (t, $J$ = 7.7 Hz, 2H), 2.47 (t, $J$ = 7.7 Hz, 2H)

Compound **2b**

2b
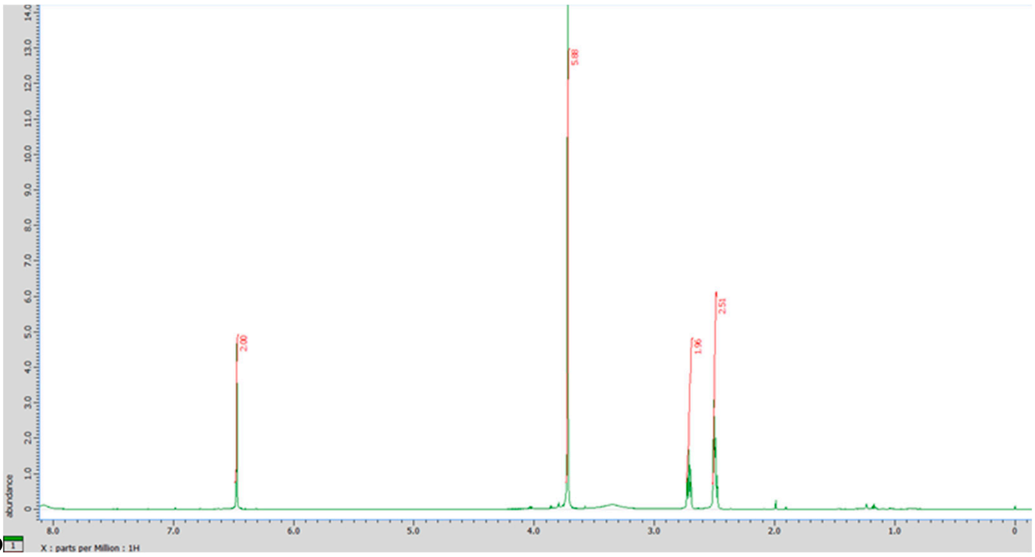


$^{1}$H-NMR (500 MHz, DMSO-D6) δ 6.47 (s, 2H), 3.72 (s, 6H), 2.71 (t, $J$ = 7.7 Hz, 2H), 2.50-2.49 (t, 3H)

Compound **3a**

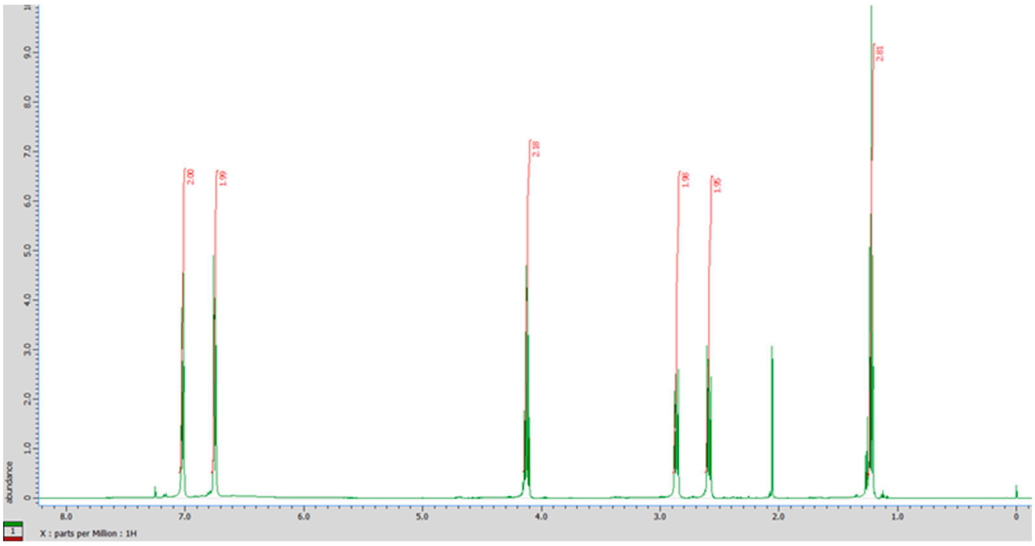

$^{1}$H-NMR (500 MHz, CHLOROFORM-D) δ 7.02 (d, *J* = 8.0 Hz, 2H), 6.75 (d, *J* = 8.6 Hz, 2H), 4.12 (q, *J* = 7.1 Hz, 2H), 2.86 (t, *J* = 7.7 Hz, 2H), 2.59 (t, *J* = 7.7 Hz, 2H), 1.22 (t, *J* = 7.2 Hz, 3H)

Compound **3b**

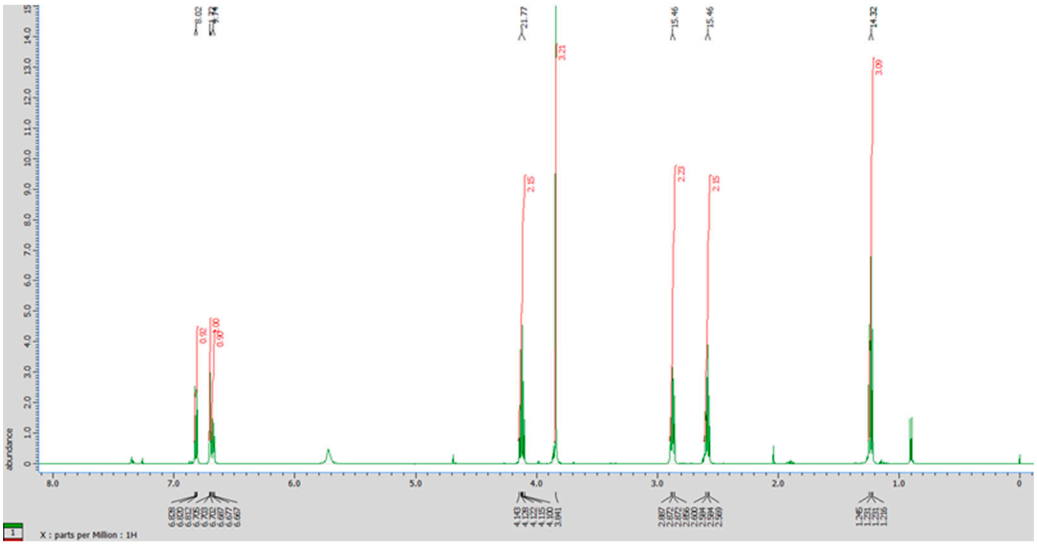

$^{1}$H-NMR (500 MHz, CHLOROFORM-D) δ 6.82 (d, *J* = 8.0 Hz, 1H), 6.70 (d, *J* = 1.7 Hz, 1H), 6.68 (d, *J* = 9.7 Hz, 1H), 4.12 (q, *J* = 7.3 Hz, 2H), 3.84 (s, 3H), 2.87 (t, *J* = 7.7 Hz, 2H), 2.58 (t, *J* = 7.7 Hz, 2H), 1.23 (d, *J* = 14.3 Hz, 3H)

Compound **3c**

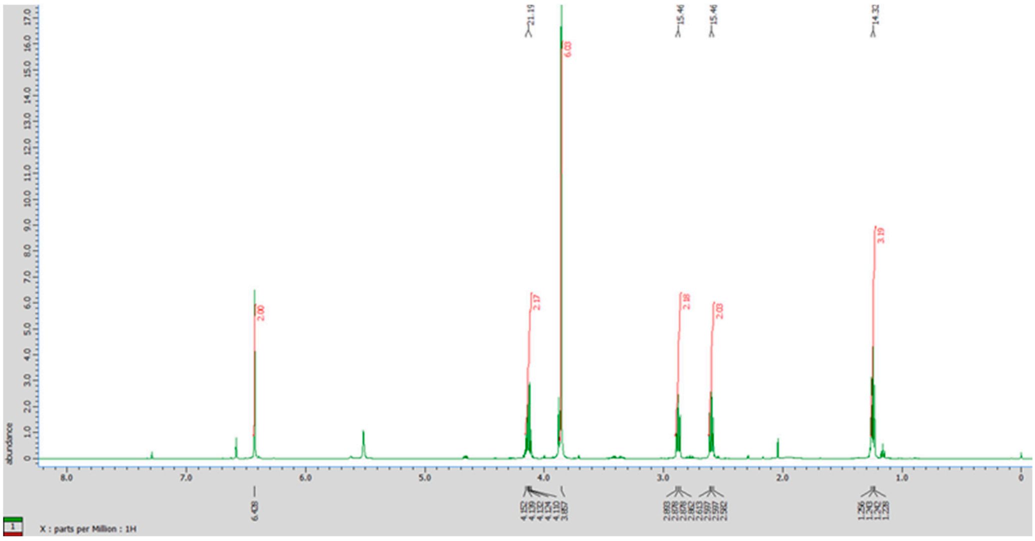

$^{1}$H-NMR (500 MHz, CHLOROFORM-D) δ 6.43 (s, 2H), 4.13 (q, *J* = 7.1 Hz, 2H), 3.86 (s, 6H), 2.88 (t, *J* = 7.7 Hz, 2H), 2.60 (t, *J* = 7.7 Hz, 2H), 1.24 (t, *J* = 7.2 Hz, 3H)

Compound **3d**

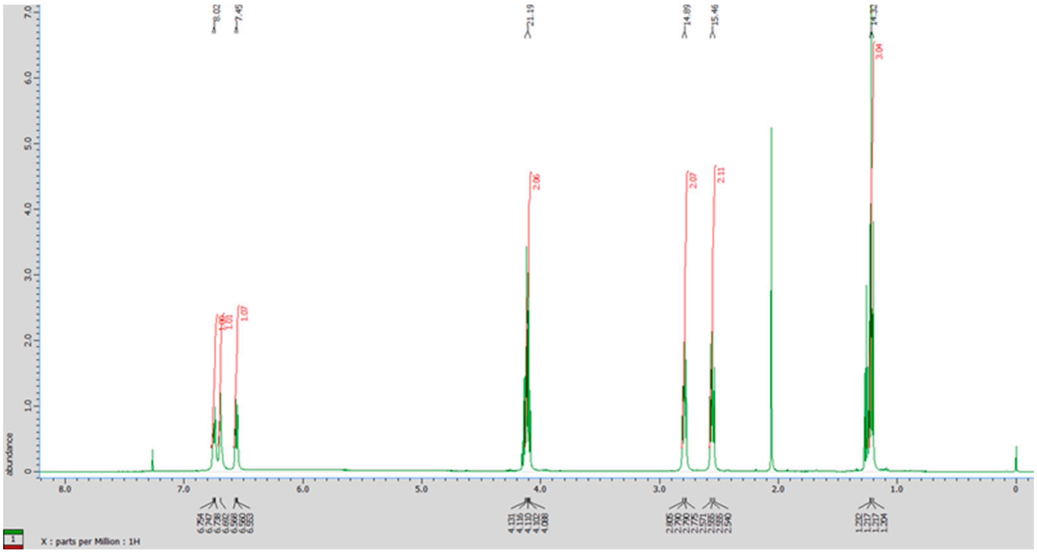

$^{1}$H-NMR (500 MHz, CHLOROFORM-D) δ 6.75 (d, *J* = 8.0 Hz, 1H), 6.69 (s, 1H), 6.56 (d, *J* = 7.4 Hz, 1H), 4.11 (q, *J* = 7.1 Hz, 2H), 2.79 (t, *J* = 7.4 Hz, 2H), 2.56 (t, *J* = 7.7 Hz, 2H), 1.22 (t, *J* = 7.2 Hz, 3H)

Compound **4a**

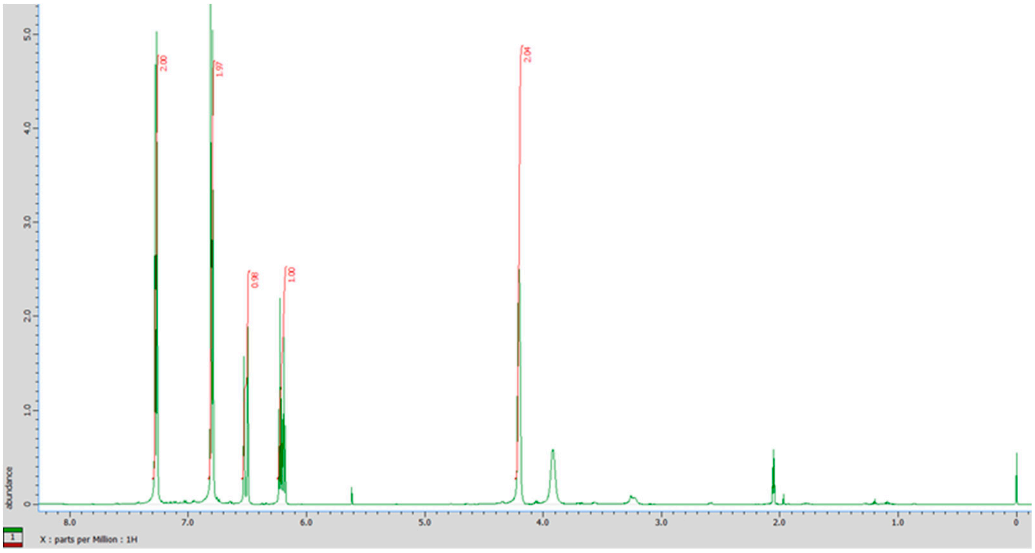

$^1$H-NMR (500 MHz, ACETONE-D6) δ 7.27 (d, *J* = 8.6 Hz, 2H), 6.80 (d, *J* = 8.6 Hz, 2H), 6.51 (d, *J* = 16.0 Hz, 1H), 6.20 (td, *J* = 10.7, 5.2 Hz, 1H), 4.20 (d, *J* = 5.7 Hz, 2H)

Compound **4b**

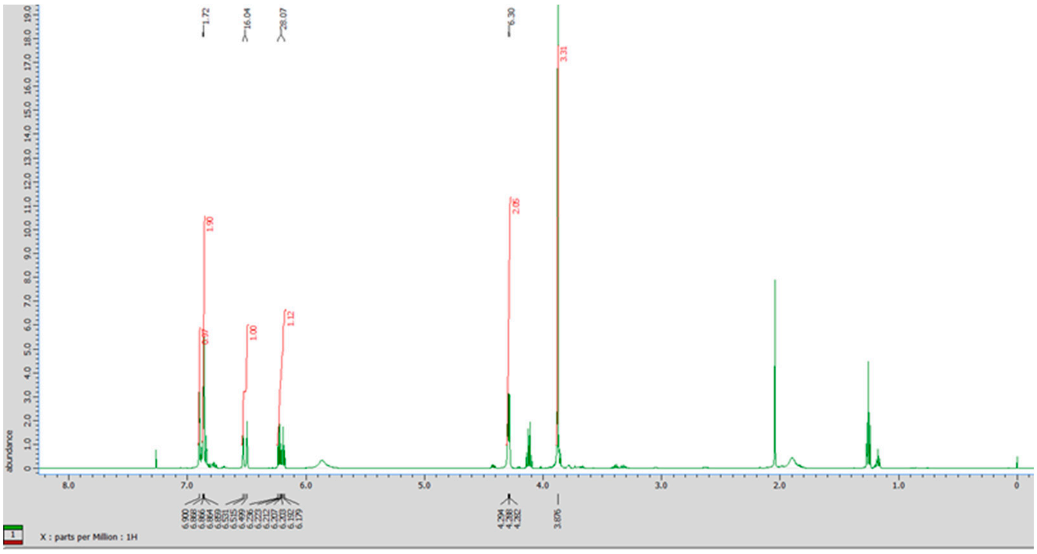

$^1$H-NMR (500 MHz, CHLOROFORM-D) δ 6.90 (s, 1H), 6.86 (t, *J* = 2.3 Hz, 2H), 6.51 (d, *J* = 16.0 Hz, 1H), 6.21 (dt, *J* = 15.8, 6.0 Hz, 1H), 4.29 (d, *J* = 6.3 Hz, 2H), 3.88 (s, 3H)

Compound **4c**

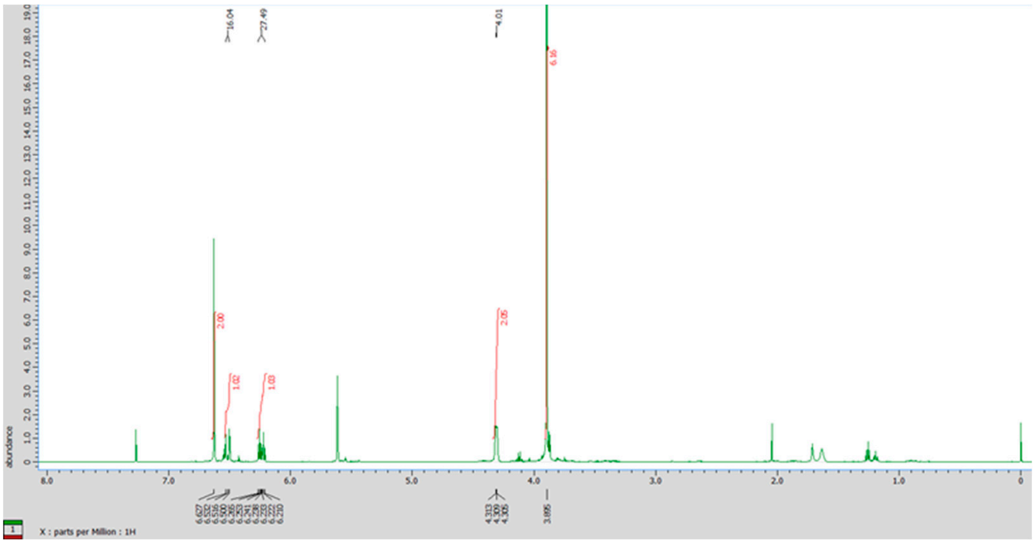

$^{1}$H-NMR (500 MHz, CHLOROFORM-D) δ 6.63 (s, 2H), 6.52 (d, *J* = 16.0 Hz, 1H), 6.24 (dt, *J* = 15.7, 5.9 Hz, 1H), 4.31 (d, *J* = 4.0 Hz, 2H), 3.90 (s, 6H)

Compound **5a**

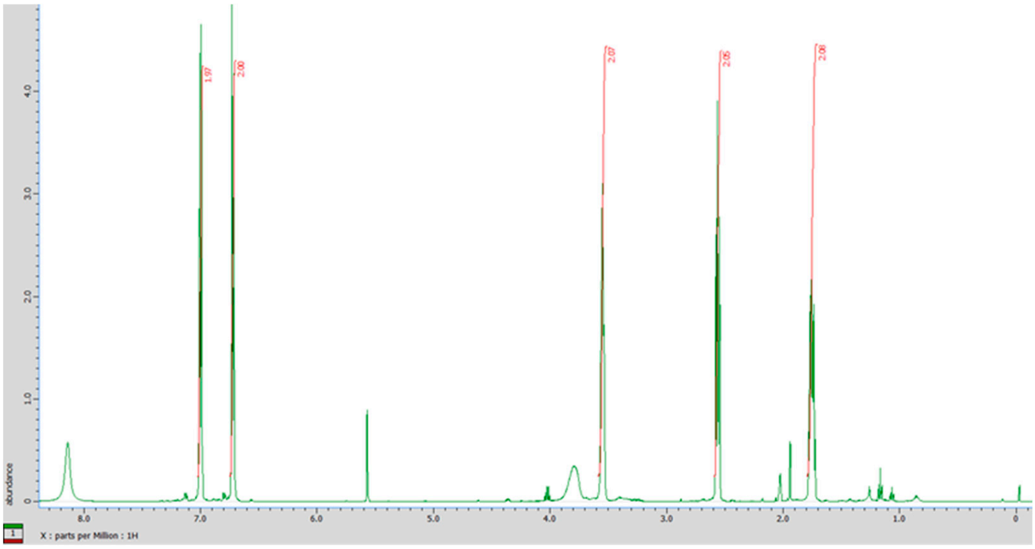

$^{1}$H-NMR (500 MHz, CHLOROFORM-D) δ 7.05 (d, *J* = 8.0 Hz, 2H), 6.75 (d, *J* = 7.4 Hz, 2H), 3.67 (t, *J* = 6.3 Hz, 2H), 2.63 (t, *J* = 7.7 Hz, 2H), 1.89-1.83 (m, 2H)

Compound **5b**

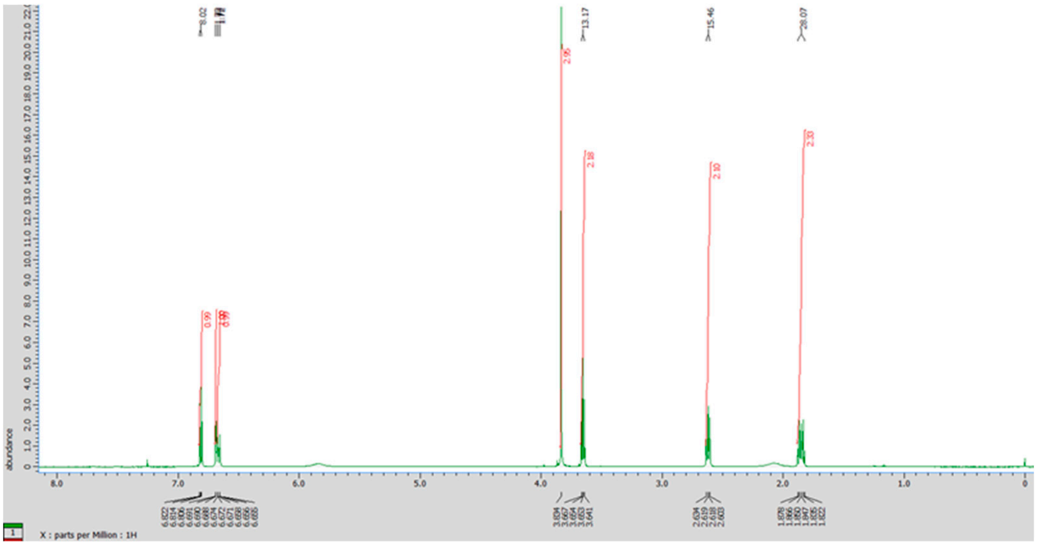

$^{1}$H-NMR (500 MHz, CHLOROFORM-D) δ 6.81 (d, *J* = 8.0 Hz, 1H), 6.69 (d, *J* = 1.7 Hz, 1H), 6.66 (dd, *J* = 8.0, 1.7 Hz, 1H), 3.83 (s, 3H), 3.65 (t, *J* = 6.6 Hz, 2H), 2.62 (t, *J* = 7.7 Hz, 2H), 1.88-1.82 (m, 2H)

Compound **5c**

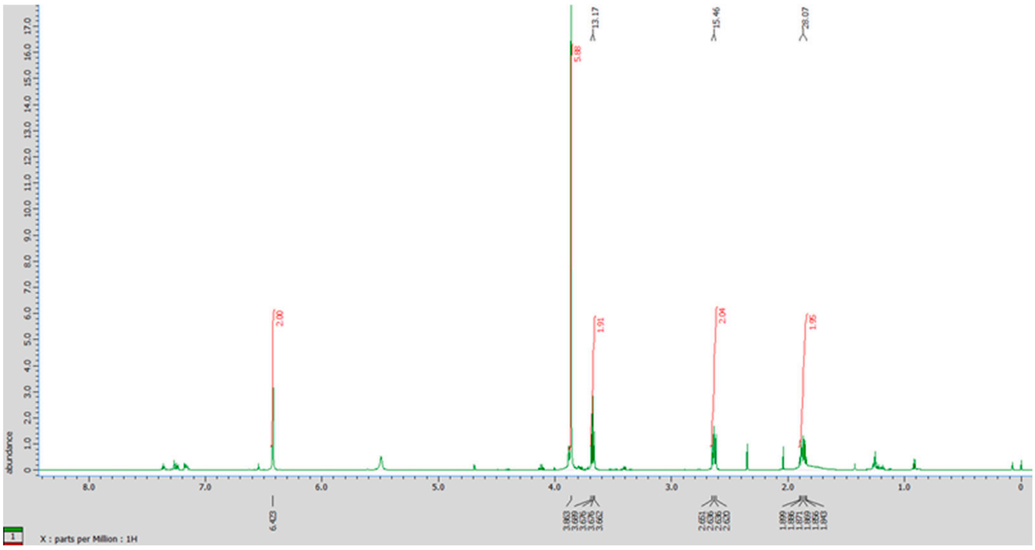

$^{1}$H-NMR (500 MHz, CHLOROFORM-D) δ 6.42 (s, 2H), 3.86 (s, 6H), 3.68 (t, *J* = 6.6 Hz, 2H), 2.64 (t, *J* = 7.7 Hz, 2H), 1.90-1.84 (m, 2H)

Compound **6a**

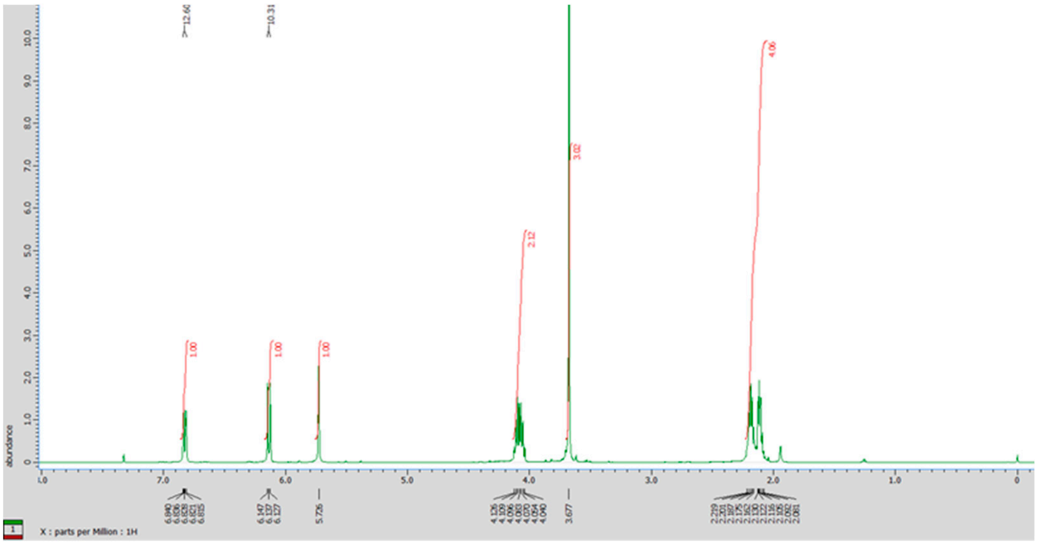

$^{1}$H-NMR (500 MHz, CHLOROFORM-D) δ 6.83 (dd, *J* = 10.0, 2.6 Hz, 1H), 6.14 (d, *J* = 10.3 Hz, 1H), 5.73 (s, 1H), 4.13-4.04 (m, 2H), 3.68 (s, 3H), 2.22-2.08 (m, 4H)

Compound **6b**

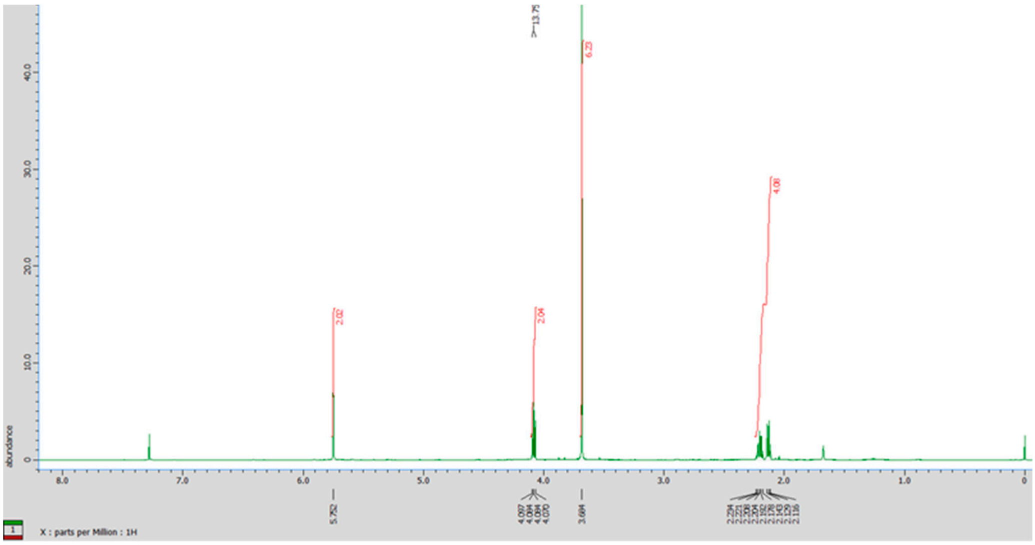

$^{1}$H-NMR (500 MHz, CHLOROFORM-D) δ 5.75 (s, 2H), 4.08 (t, *J* = 6.9 Hz, 2H), 3.68 (s, 6H), 2.23-2.12 (m, 4H)

Compound **7b**(Dehydrodiconiferyl Alcohol)

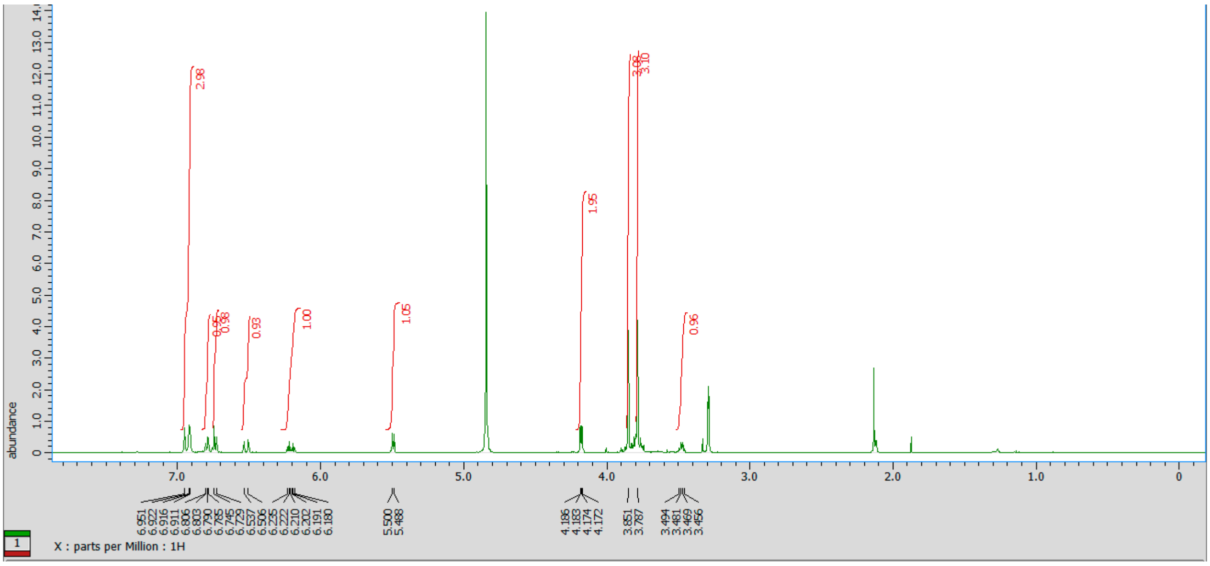


$^{1}$H-NMR (500 MHz, METHANOL-D3) δ 6.95-6.91 (m, 3H), 6.80 (dd, *J* = 8.3, 2.0 Hz, 1H), 6.74 (d, *J* = 8.0 Hz, 1H), 6.52 (d, *J* = 15.5 Hz, 1H), 6.21 (dt, *J* = 15.7, 5.9 Hz, 1H), 5.49 (d, *J* = 6.3 Hz, 1H), 4.18 (dd, *J* = 5.7, 1.1 Hz, 2H), 3.85 (s, 3H), 3.79 (s, 3H), 3.48 (q, *J* = 6.3 Hz, 1H)

Compound **8a**

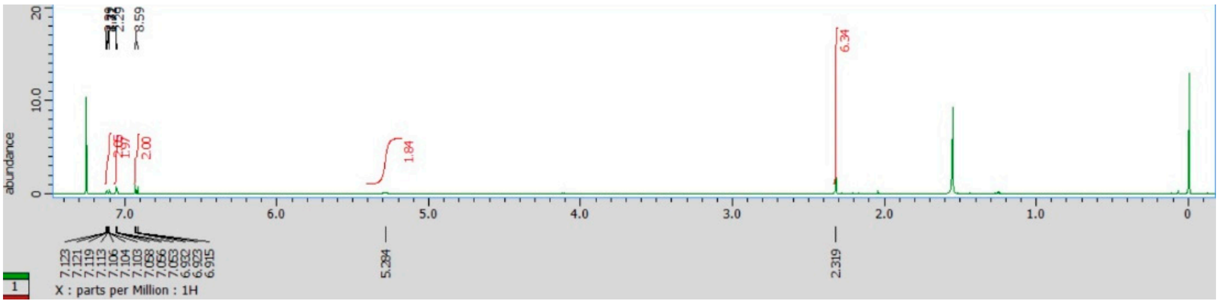


$^{1}$H-NMR (500 MHz, CHLOROFORM-D) δ 7.11 (dd, *J* = 8.3, 2.0 Hz, 2H), 7.06 (d, *J* = 2.3 Hz, 2H), 6.92 (d, *J* = 8.6 Hz, 2H), 5.28 (br, 2H), 2.32 (s, 6H)

Compound **8b**

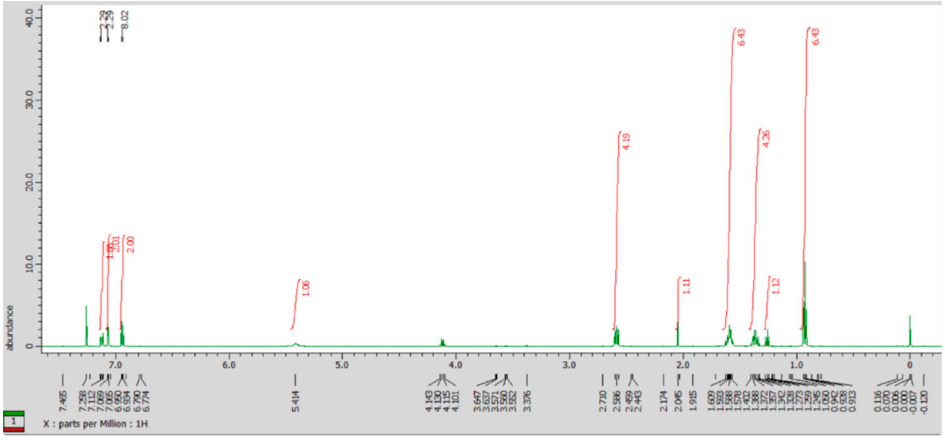

$^1$H-NMR (500 MHz, CHLOROFORM- d) δ = 7.12 (dd, *J* = 8.0, 2.3 Hz, 2H), 7.07 (d, *J* = 2.3 Hz, 2H), 6.94 (d, *J* = 8.0 Hz, 2H), 2.59 (t, *J* = 7.5 Hz, 4H), 1.56-1.62 (m, 4H), 1.37 (sext, *J* = 6.9 Hz, 3H), 0.93 (t, *J* = 7.5 Hz, 6H)

Compound **8c**

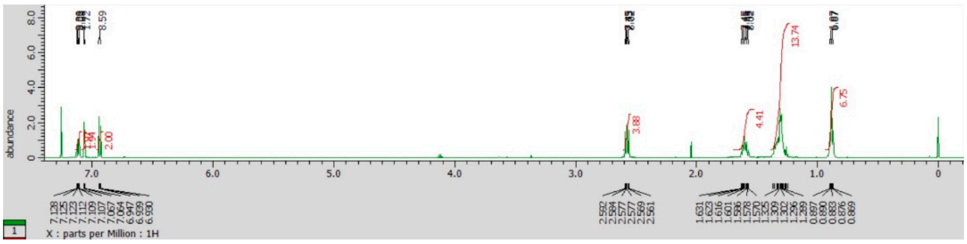

$^1$H-NMR (500 MHz, CHLOROFORM-D) δ 7.12 (dd, *J* = 8.0, 2.3 Hz, 2H), 7.07 (d, *J* = 1.7 Hz, 2H), 6.94 (d, *J* = 8.6 Hz, 2H), 2.58 (t, *J* = 7.7 Hz, 4H), 1.60 (quins, *J* = 7.6 Hz, 4H), 1.37-1.24 (m, 12H), 0.88 (t, *J* = 6.9 Hz, 6H)

Compound **8d**

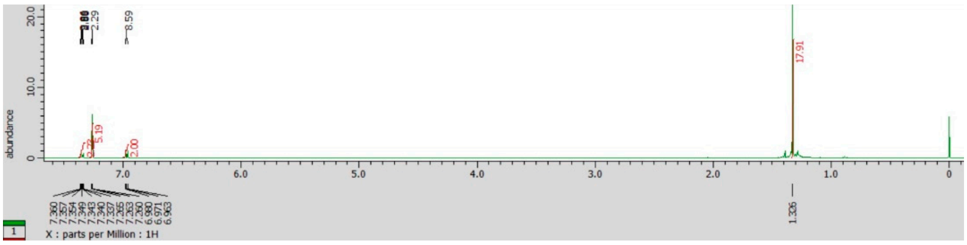

$^1$H-NMR (500 MHz, CHLOROFORM-D) δ 7.35 (dd, *J* = 8.6, 2.9 Hz, 2H), 7.26 (d, *J* = 2.3 Hz, 2H), 6.97 (d, *J* = 8.6 Hz, 2H), 1.33 (s, 18H)

Compound **8e**

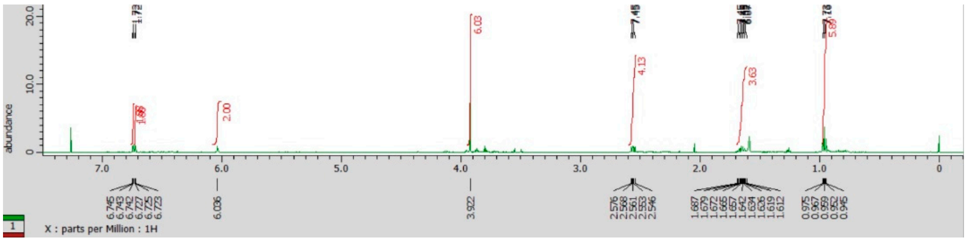


$^1$H-NMR (500 MHz, CHLOROFORM-D) δ 6.74 (d, J = 1.7 Hz, 2H), 6.73 (d, J = 1.7 Hz, 2H), 6.04 (s, 2H), 3.92 (s, 6H), 2.56 (t, J = 7.4 Hz, 4H), 1.65 (td, J = 15.0, 7.3 Hz, 4H), 0.96 (t, J = 7.4 Hz, 6H)

Compound **9a**

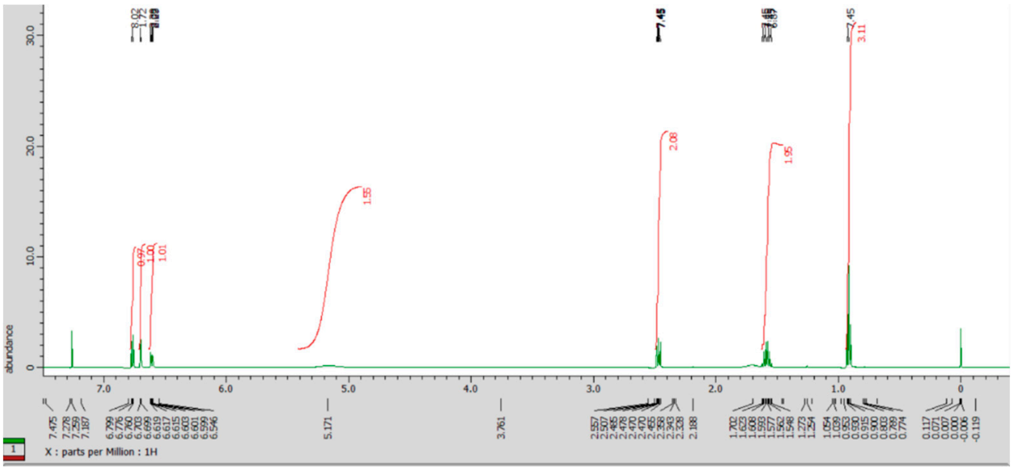


$^1$H-NMR (500 MHz, CHLOROFORM- d) δ = 6.77 (d, *J* = 8.0 Hz, 1H), 6.70 (d, *J* = 1.7 Hz, 1H), 6.61 (dd, *J* = 8.0, 2.3 Hz, 1H), 2.47 (t, *J*=7.5 Hz, 2H), 1.59 (sext, *J* = 7.5 Hz, 2H), 0.92 (t, *J* = 7.5 Hz, 3H)

Compound **9b**

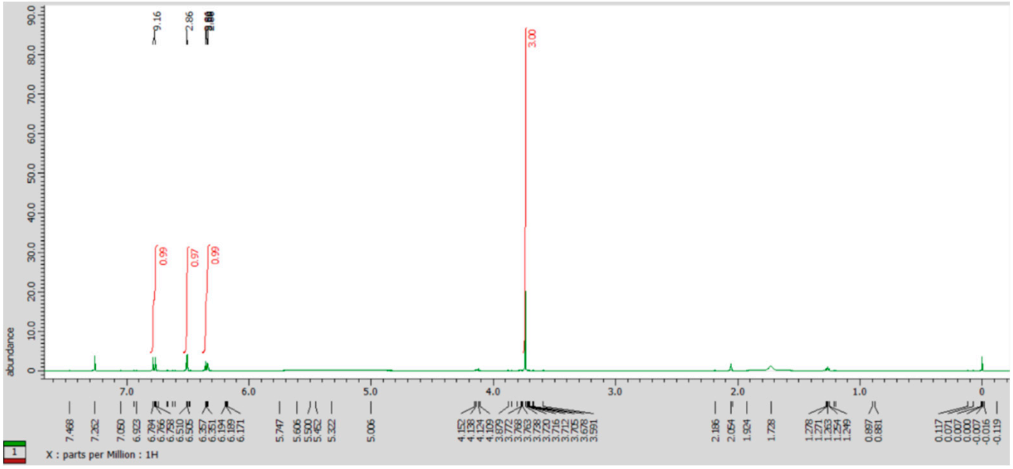

$^{1}$H-NMR (500 MHz, CHLOROFORM- d) δ = 6.78 (d, *J* = 9.2 Hz, 1H), 6.51 (d, *J* = 2.9 Hz, 1H), 6.35 (dd, *J* = 8.6, 2.9 Hz, 1H), 3.74 (s, 3H)
