# Supplementary material for: DPPH Measurements and Structure—Activity Relationship Studies on the Antioxidant Capacity of Phenols
Source: Antioxidants (Basel). 2024 Mar 1;13(3):309. doi: 10.3390/antiox13030309 (PMC10967577; doi:10.3390/antiox13030309)
Supplement: Supplementary file 1 [file antioxidants-13-00309-s001.zip › SI.pdf]

DPPH measurements and structure-activity relationship studies of the antioxidant capacity of phenols

Moeka Yamauchi, Yukino Kitamura, Haruka Nagano, Junya Kawatsu, Yurika Ito and Hiroaki Gotoh*

Contents

1. Method of measuring DPPH 2

2. Calculation of semi-empirical quantum chemical calculation values 3

2.1 Data processing and calculations for FOODB 3

2.2 Structure Conformational search 3

2.3 MOPAC2016(PM7) calculation 3

3. Supplementary experimental and calculation data analysis 4

3.1 Mean and standard deviation of DPPH test measurements 4

3.2 Scatter plot matrix of calculated values 5

Attached File List

1. DPPH measured value file:
   TEAC_measured_with_DPPH_test_in_the_lab.xlsx
2. TEAC in the literature:
   TEAC_measured_with_the_same_kit_reported_in_literatures.xlsx
3. Comparison with H-ORAC value:
   measured_H_ORAC_to_compare_with_DPPH.csv
4. Calculation of phenols in FOODB:
   calculation_result_of_phenols_in_FOODB.csv
5. Synthetic method, file of composite spectra:
   SI(synthesis).docx

1. Method of measuring DPPH

The DPPH Antioxidant Assay Kit (FUJIFILM Wako Pure Chemical Corporation) was used for the assay. Measurements were performed according to this manual of this kit. 96-well microplates (Thermo Scientific Nunc), a multispectro microplate reader Varioskan Flash (Thermo Fischer Scientific) were used. Ethanol was used as the solvent. TEAC was determined in this experiment after obtaining the optimum concentration range for each compound as a preliminary experiment. IC_{50} is defined as the sample concentration that reduces the DPPH concentration by 50% after 30 minutes of reaction. The ratio of volumes of sample solution, assay buffer and DPPH solution was 4:1:5 and the reaction was performed at 25°C. TEAC is defined as in the following equation.

$$TEAC = \frac{IC_{50}\ of\ trolox}{IC_{50}\ of\ sample}$$

The IC_{50} was determined by measuring the DPPH concentration before and after the reaction by measuring the absorbance at the maximum absorption wavelength of DPPH radicals, 517 nm. To each well of the microplate, 20 μL of sample, 80 μL of assay buffer, and 100 μL of DPPH solution were added in sequence. The wells were placed in a thermostatic bath at 25 °C for 30 minutes, and the absorbance was measured at 517 nm by a microplate reader. A sample blank and a DPPH solution blank were also prepared; ethanol was added in place of sample to the sample blank, and ethanol was added in place of sample and DPPH solution to the DPPH solution blank. The post-reaction DPPH scavenging rate and IC_{50} for each solution were calculated as in the equation below.

$$R_{DPPH} = \frac{\left(Abs_{sample} - Abs_{blank(ethanol)}\right) - \left(Abs_{blank(DPPH\ solution)} - Abs_{blank(ethanol)}\right)}{\left(Abs_{blank(DPPH\ solution)} - Abs_{blank(ethanol)}\right)}$$

The DPPH scavenging rate was measured at four or more locations where the DPPH scavenging rate was close to 50, and linear regression was performed on four or more points where linearity was observed in the regression line to determine the sample concentration at which the DPPH scavenging rate was 50 %. The standard deviation was determined for each sample and blank concentration by performing the same reaction in three wells. The IC_{50} was not measured for samples above 1000 μg/mL. The stoichiometry number was calculated as the number of DPPH radicals scavenged per molecule of antioxidant in the reaction at 50% inhibitory concentration.

2. Calculation of semi-empirical quantum chemical calculation values

2.1 Data processing and calculations for FOODB

All FOODB compound data including SMILES were downloaded in February 2023; non-neutral molecules with + or - in SMILES were excluded; the number of aromatic hydroxy groups was calculated by the RDKit method fr_Ar_OH and compounds with 0 were excluded. Quantum chemical calculations were performed only for IP using the same method as in Section 2. Those compounds for which the MOPAC2016(PM7) calculation was not completed successfully were excluded. Finally, calculated values were obtained for 4240 compounds.

2.2 Structure Conformational search

The procedure for searching for neutral molecular conformers is described below. 1000 conformers were generated based on the distance diometry method, each conformer was optimized under the MMFF force field, and the most stable of the optimized conformers was selected. All these operations were performed using RDKit. The functions and arguments used in the conformational search are listed below. However, useRandomCoords=True was used for tannic acid, which has a large molecular weight. Neutral molecules, radicals, radical cations, and anions were all calculated using the conformers of this searched neutral molecule as the initial conformation. For radicals and anions, dissociated radicals and anions were calculated for all hydrogen atoms in the molecule, and the most stable conformations were employed for PA, ETE, PDE, and BDE calculations.

Conformational search

```
cids = AllChem.EmbedMultipleConfs(mol, numConfs=1000, randomSeed=1234,
                                  pruneRmsThresh=1, numThreads=0)
```

structural optimization

```
AllChem.MMFFGetMolecularProperties()
AllChem.MMFFGetMolcularForceField()
```

2.3 MOPAC2016(PM7) calculation

MOPAC2016(PM7) was used for the calculations. The keywords used in the calculations for each of the neutral molecules, radicals, radical cations, and anions are listed below.

Table S1

	keyword
neutral	PM7 EF PRECISE GNORM=0.05 NOINTER GRAPHF VECTORS MMOK
radical	PM7 EF PRECISE GNORM=0.05 NOINTER GRAPHF VECTORS DOUBLET MMOK

radical cation	PM7 EF PRECISE GNORM=0.05 NOINTER GRAPHF VECTORS DOUBLET CHARGE=1 MMOK
anion	PM7 EF PRECISE GNORM=0.05 NOINTER GRAPHF VECTORS CHARGE=-1 MMOK

IP, BDE, PDE, PA, and ETE were calculated from equations (1) through (5) below. For anions and radicals, BDE and PA, which have the lowest calculated heat of formation in the molecule, were used. The values of H(e-) were quoted in vacuum [42]. For the compounds extracted from FOODB (4240 species) and those whose DPPH was measured in the literature [30], [31], [32], [33], [34], [35], [36] (24 species), only IP was calculated according to the same method. Details of the calculation method are described in SI.

$$\mathrm{BDE} = \mathrm{H}(\mathrm{Ar}O^{\cdot}) + \mathrm{H}(\mathrm{H}^{\cdot}) - \mathrm{H}(\mathrm{ArOH}) \quad (1)$$

$$\mathrm{IP} = \mathrm{H}(\mathrm{ArOH}^{\cdot+}) + \mathrm{H}(e^{-}) - \mathrm{H}(\mathrm{ArOH}) \quad (2)$$

$$\mathrm{PDE} = \mathrm{H}(\mathrm{ArO}^{\cdot}) + \mathrm{H}(\mathrm{H}^{+}) - \mathrm{H}(\mathrm{ArOH}^{\cdot+}) \quad (3)$$

$$\mathrm{PA} = \mathrm{H}(\mathrm{ArO}^{-}) + \mathrm{H}(\mathrm{H}^{+}) - \mathrm{H}(\mathrm{ArOH}) \quad (4)$$

$$\mathrm{ETE} = \mathrm{H}(\mathrm{ArO}^{\cdot}) + \mathrm{H}(\mathrm{e}^{-}) - \mathrm{H}(\mathrm{ArO}^{-}) \quad (5)$$

3. Supplementary experimental and calculation data analysis

3.1 Mean and standard deviation of DPPH test measurements

Table S2 Catechin

Entry	IC_{50} [µg/mL]	TEAC [molTE/mol]
0	27.01	2.39
1	28.01	2.10
2	32.39	1.86
3	33.51	1.92
4	18.52	2.95
5	26.65	2.08
6	26.59	2.38
7	31.21	2.06
8	29.79	1.96
9	37.13	2.22
10	23.42	2.67
mean	28.57	2.24
Standard deviation	5.09	0.34

Table S3 trolox

Entry	IC_{50} [μg/mL]
0	65.00
1	69.56
2	64.43
3	58.70
4	64.43
5	58.70
6	64.47
7	62.57
8	69.56
9	65.00
10	60.34
11	64.40
12	53.85
13	55.48
14	63.14
15	64.15
16	58.43
17	82.32
18	66.98
19	61.56
20	68.64
21	59.46
22	68.64
mean	63.90
Standard deviation	5.87

3.2 Scatter plot matrix of calculated values

The results of various calculations were analyzed for overall trends. A scatter plot matrix of the calculated values is shown in Figure S2. The blue dots are for compounds with TEAC greater than 0.2 [molTE/mol] and the orange dots are for compounds with TEAC less than 0.2 [molTE/mol]. Tannic acid with a TEAC of 15.4 [molTE/mol] is excluded for visibility. Looking at the group of compounds measured, correlations were observed between IP and PDE and between PA and ETE (Table 1), with a weak correlation (correlation coefficient 0.53) between IP and BDE. The correlations between IP and PDE (correlation coefficient -0.86) and between PA and ETE are due to the fact that they are indicators concerning one reaction pathway and are highly dependent on the energy of unstable structures in the reaction pathway. Weak correlations were

also observed between IP and BDE because the stability of both phenoxy radicals and radical cations depends on the electronic state of the benzene ring.

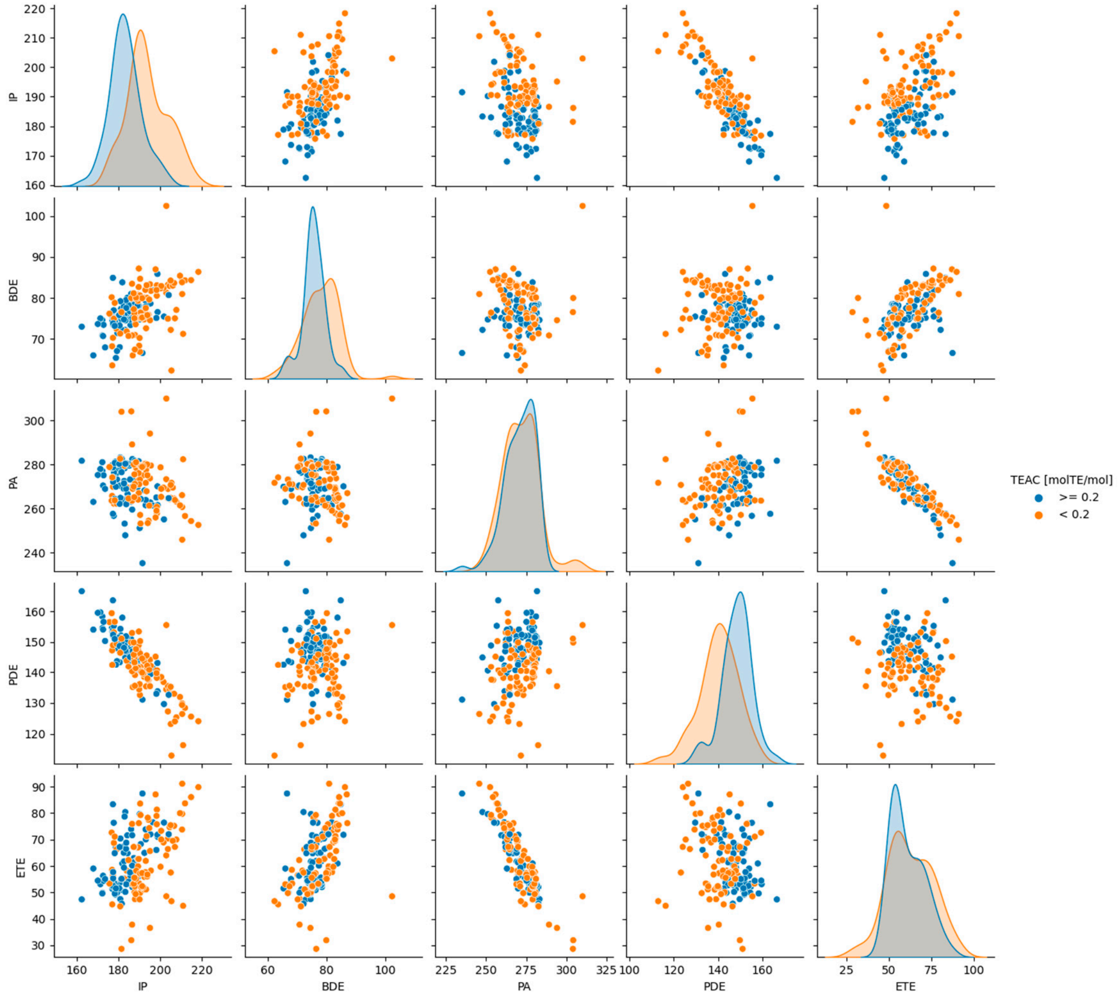

Figure S1 Scatter plots matrix of calculated values by MOPAC2016 (PM7)) of compounds measured in the lab. Scatter plots between different calculated values and diagonal distribution plots. Blue dots are compounds with TEAC greater than 0.2 [molTE/mol]. Orange points are for compounds with TEAC less than 0.2 [molTE/mol]. Proton Affinity (PA)、Electron Transfer Enthalpy (ETE)、Bonding Dissociation Enthalpy (BDE)、Proton Dissociation Enthalpy (PDE)、Ionization Potential (IP). All units are [kcal/mol]. This plot was drawn by the seaborn library.

Table S4 Correlation coefficients for IP, BDE, PA, PDE, and ETE values calculated in MOPAC2016 (PM7).

	IP	BDE	PA	PDE	ETE
IP	1.00	0.50	-0.27	-0.86	0.46
BDE	0.50	1.00	-0.03	0.02	0.47
PA	-0.27	-0.03	1.00	0.30	-0.89

PDE	-0.86	0.02	0.30	1.00	-0.25
ETE	0.46	0.47	-0.89	-0.25	1.00

Table S5 Mean and standard deviation for active (TEAC [molTE/mol] >= 0.2) and inactive (TEAC [molTE/mol] < 0.2) compounds.

	Mean (active)	Std (active)	Mean (inactive)	Std (inactive)
IP	182.22	7.32	193.42	9.60
BDE	74.29	4.38	77.96	6.27
PA	270.47	9.12	271.17	11.13
PDE	148.11	6.63	140.58	8.87
ETE	59.86	9.62	62.83	13.33

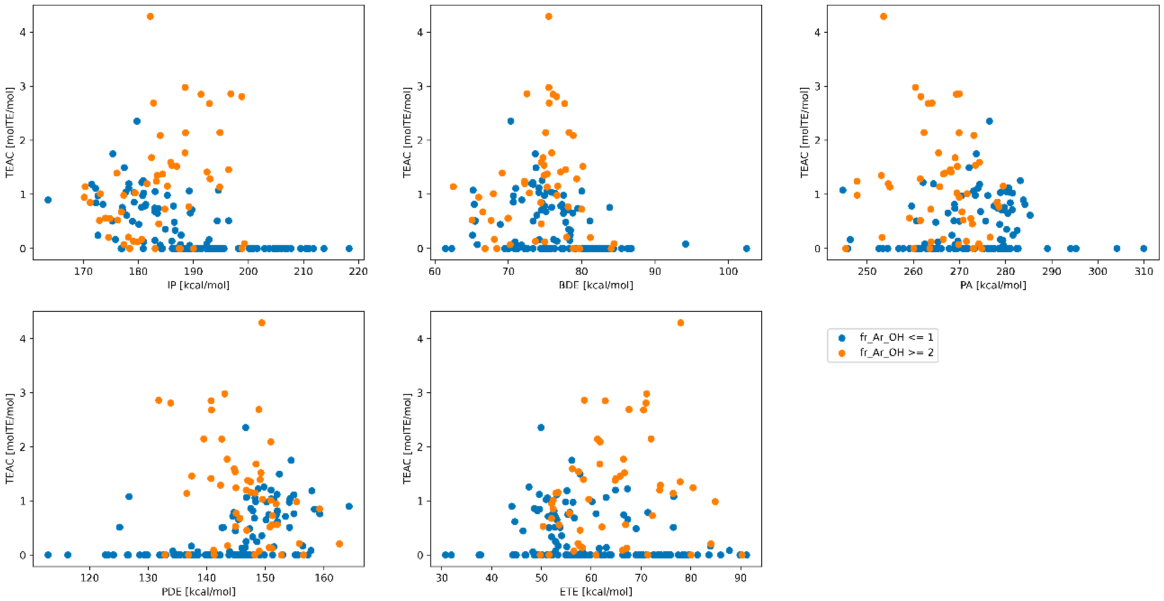

Figure S2 Relationship between the calculated values and TEAC [molTE/mol] of compounds measured in the lab. Colors are assigned according to the number of aromatic hydroxy groups. Blue: aromatic hydroxy groups less than 2, red: aromatic hydroxy groups more than 1. This scatter plot was drawn by matplotlib library.

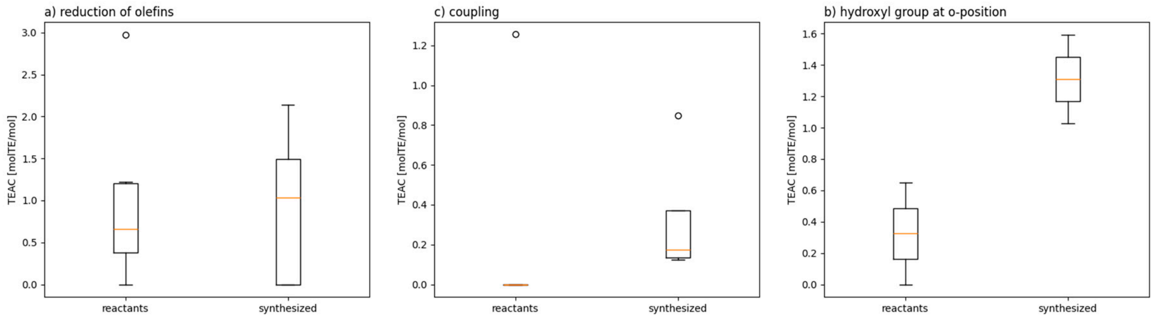

Figure S3 Box-and-whisker diagram of TEAC values in the DPPH test for post-synthesized (synthesized) and pre-synthesized (reactants) compounds. a) reduction of an olefin at the benzyl position b) coupling reaction, c) introduction of a hydroxy group at the ortho position. This diagram was drawn by matplotlib library.
